# The Grading of Osteogenic Sarcoma

**DOI:** 10.1038/bjc.1952.5

**Published:** 1952-03

**Authors:** C. H. G. Price


					
46

THE GRADING OF OSTEOGENIC SARCOMA.

C. H. G. PRICE.

From the Medical Research Laboratory, Department of

Pathology, University of Bristol.

Received for publication December 8, 1951.

DURING the past thirty years much useful progress has been made in the
histological grading of malignant tumours of epithelial origin. This work, of
which one practical application has been the attempted correlation of prognosis
with histological structure of a tumour, has had many valuable results; and
although there may be not infrequent discrepancies between the histological
appreciation and the outcome of treatment in any given type of neoplasm, the
results have at any rate led to some attempt being made by the histo-pathologist
to guide the clinician at least in qualitative terms of high or low malignancy;
and to more precise premises for the comnparison of differing methods of treat-
ment in series of comparable cases.

The starting point of this present analysis was the observation that irrespective
of the mode of treatment applied, the overall five year survival rates in most
published dissections of groups of cases of osteogenic sarcomata were approxi-
nmately of a similar order. A recent exception to this is contained in the analysis
of a group of 415 malignant tumours involving bone published by Prevo (1950).
This large series included a group of 41 cases of osteogenic sarcoma confirmed by
biopsy in which there were two 5-year survivals (4.9 per cent). The mean duration
of symptoms prior to treatment in this series was 8- 6 months, which is rather
prolonged. In the small group in the Bristol Bone Tumour Register the mean
time from the onset of symptoms to the time of clinical and radiological diagnosis
was 4 4 months, and in the British Empire Cancer Campaign analysis (1949) it
was shown that 69- 9 per cent of all cases attended for their first consultation in
1 to 3 months from the time of onset of symptoms. In Prevo's (1950) series the
average duration of symptoms to the time of death was 22 6 months, in the
Bristol Bone Tumour Register group here reported this period was rather shorter-
18*1 months.

Prevo (1950) comments that the 5-year survival rate in his series does not
agree with those published by Geschickter and Copeland (1949) and others, but
makes no suggestion to account for the marked discrepancy.

Although the number of cases of osteogenic sarcoma available in the Bristol
Bone Tumour Register collection is still small (36), it will presently be shown that
in broad outline the series conforms with the general features more clearly de-
lineated by the examination of much larger numbers of cases. It was therefore
thought useful to subject the clinical, radiological and histological records of the
36 cases to a close examination with a view to the establishment of a means of
grading these tumours on their histological structure, and to determine to what
extent the biological history of each particular tumour could be matched with the
microscopic picture.

GRADING OP OSTEOGENIC SARCOMA

It is noteworthy that two of the most valuable general works in this field-
Geschickter and Copeland's 'Tumours of Bone' (1949) and Coley's 'Neoplasms of
Bone' (1949)-make no mention of histological grading of the tumours except in
general terms, and although the latter subdivides cases into those of low, average
and high malignancy, there is but little to indicate the histological features by
which the differentiation can be made, other than the examination of cell mor-
phology and stromal character. It is not infrequently the fashion to subdivide
osteogenic sarcoma into a number of types-medullary and sub-periosteal, telan-
giectatic, sclerosing, periosteal, fibrosarcoma, chondrosarcoma, etc. This does
not always seem profitable, and the writer is in accord with the opinion expressed
by Willis (1948) on this point. Chondrosarcoma appears to differ in several major
ways from the typical osteolytic anid osteoblastic osteogenic sarcoma. In its
age incidence, main sites of origin, behaviour, its radiological and macroscopic
appearances it appears to be characteristic, although in a broader sense it may be
regarded as a variant of the osteogenic sarcoma group. For this reason the cases
of chondrosarcoma in the Bristol Bone Tunmour Register have not been included
in the series of tumours here examined.

On perusal of the literature on osteogenic sarcoma it appears that where other
attempts at histological grading have been made, e.g., Simmons (1939), here again
this has mainly been done on considerations of cell type and stromal characters.
It is probable that these writers have been influenced by the approach of histo-
logists to the group of fibro-sarcomata, where again the main histological grading
has been based almost entirely upon cell morphology and the amount of stromal
material produced by the neoplastic cells. This would seem to be the case in the
grading of sarcomata adopted in such papers as those of Quick and Cutler (1927),
Grynfeltt (1929), Geschickter (1932), and Broders, Hargrave and Meyerding (1939)
to mention a representative few.

The present position may be more clearly shown by quoting from a paper by
Coley and Pool (1940): " Microscopically, the grade of malignancy is demon-
strable by a uniformity of pleomorphism of the cell type, and we feel that in a
given case, the most highly malignant area seen under the microscope should
determine the grade". The latter point is undoubtedlv true, but it is suggested
that the fundamental factor which dominates the selection of the " most highly
malignant area" is only appreciated in a general way, i.e., the degree of mitotic
activity of the neoplastic cells. It is a pathological truism to state that the
presence of numerous mitotic nuclei in a section with concomitant atypism is
indicative of a high degree of malignancy. The main purpose of this present
paper is to define this well-appreciated fact in more precise terms and to focus
attention more clearly upon its significance. Coley and Pool (1940) rightly draw
attention to the other factors of a gross pathological nature which should influence
the decision as to the grading of any particular sarcoma. The inspection of
macroscopic pathology must largely be consequent upon either open operation,
amputation or autopsy, and such details are not likely to be available to the
histologist at the time of diagnosis. Some of these features can, it is true, be
visualised by X-ray plates. However, the interpretation of these is mainly the
proper province of the radiologist, and they are seldom to be seen in the routine
pathological laboratory. For the fortunate histo-pathologist who may be granted
access to such aids, the position is only inmproved to such an extent as the experi-
ence which he may have in the interpretation of skiagrams, which is a field with

47

48

many pitfalls. The ideal state no doubt is the close liaison between surgeon,
radiodiagnostician, radiotherapist and histologist with personal conference in
each and every case. Such states of collaboration seldom exist under conditions
of normal routine work.

The histological specimens from the present series of cases have been considered
under the following headings:

1. Predominant cell type and its morphological characteristics; the
type and amount of concomitant stroma.

2. The degree of nuclear mitotic activity present in selected areas of
each sarcoma.

MATERIALS AND METHODS.

The cases comprise those which have been referred to the Bone Tumour
Register during the 5-year-period 1946 to 1951. To anyone working in this field
the fallacies of diagnosis are only too well known, and the cases included have all
been subjected to free discussion and unanimous acceptance as osteogenic sarcoma
by the group of radiologists, radiotherapists, surgeons and pathologists which
forms the working panel of the Register. In each case the diagnosis has been
based upon consideration of the full history, clinical examination, adequate skia-
grams and histological sections. In some instances the histological material is
derived from biopsy, in others from surgical or autopsy specimens. This has
been indicated in Table I, also the time relationship of the histological specimen
to any pre-operative X-radiation.

For histological purposes the specimens were fixed, cut and stained by the
usual routine methods. The evaluation of the degree of mitotic activity for each
tumour has been based upon the calculation of the Mitotic Ratio (M.R.). This
is given as the ratio of resting nuclei to one cell nucleus in active mitosis, and for
the purposes of this investigation a nucleus was considered to be in mitosis from
the time of rupture of the nuclear membrane until the reformation in the cell of
two or more complete daughter nuclei. Owing to the presence in sonle sections
of many multinucleated tumour giant cells, and the indistinct outlines of a large
proportion of the polyhedral cells, the nucleus was taken as the unit rather than
the cell. In each case a minimum of 2500 neoplastic cell nuclei were examined
in each section, using a ruled internal screen in one ocular of a binocular micro-
scope. The fine ruling of this screen subdivided the central area of the observed
field into 25 equal squares, which enabled accurate counting to be performed at
a magnification of 500. In each case the examination was confined to the most
cellular areas in the sections available of each tumour, these being selected by
careful scanning with the lower power objectives.

Repeat examinations were made of a number of histological specimens with a
view to determining the degree of reliability of the method used to evaluate the
mitotic ratio of each tumour. Typical results of these counts are shown in Table
II. In each instance at least 2500 nuclei were examined.

General Features of Group of 36 Osteogenic Sarconmta.

Owing to the small number of cases available for this investigation, a brief
analysis is given for comparison with the data obtained from much larger series
of cases. Details are given in Tables I and II.

C. 1. 0. PRICV,

C. H. G. PRICE

*   4

1~~  0   &

g   'E      --

0  ~~ 4A   0  0
9          0~~

.XX E -   'o
tt 4a    '?

og
es

4-

.;
^

?

az

Pz 9

o   .

11  *

._ C

*  ee

bX 4s

4 o R-

.,.IR

0 2  o O  o     o    bo ^^              t^^       *

rU~~~~~~~~              0 o R

o i                       4-' X004400  4.'.) 0000s   0

>oR&C O _ IC;l t a  C FC  O O{ O  C) t-O O _--  C- 00   - ;  CU
,4; _     0g D ez  _ c *  ^cq  _ Ie P O cq C* m *I _q  _ s *I M4_ _

'22

EH   OaOg

0   o

aE3,bO i

:0.

fr  U s

o b O O  .

Q

ot
Cs

z

:- : :-   t-      a

^? .2 ^?  8   .g . . .^?

o=    C   r  - o

. x         oCq

44 P~; 4 od4

IfI

-- 1:4 6P4P

CD o  w :ooc -4 = C_ o-c _ o =

-   C v ctI   D  L- w C  = o

4

O    Q Ct-

ocs4

49

C. H. G. PRICE

0

S         C

01I O   ,

4D w  ^S  t

P4          P-4 t t t ;tAG- e

*49 ~ ~ ~ 5'

bo  -   4   "  0

0  Cs

;~~~~~C C_ OI d  q*  )t

0   ~ ~ ~ ~   -P ldq-m -P   a
*qb

** .

>;,. ' m ?

04       W

Pe        O:

C5

o       I

o      Co
z4      v

CC -

1 0   0
aCo- .- Z

to 10

04

S

0

0

O 0 C
^C4 10 C
^d    0

0~ 0

I      I   -   O

bO   .b   ..   4z

I2C o;

I     1

v4     P

lo    lo

ICu

I        1

. . .

.'14

I-  C   =

*

-  C  Co Cmtq eo  1000

= = 0        01 Co 1 0o
_- _- oa ot cq ez N m e

o

Z _D U

0

~0o

w04

~ 4

10

04D

d-ow
bObOO

.52 S

V ?

4 0  co5

E4~~~.;- r,

4-S

40

5, g EEr

o 8

* . ~ .  SS

0~~  Co Co C . C

50

&6
'4

0-

'~4
0

0 *
: ,~ t

b0Q C. :0t

I        I   I
m I       00

;40? ?dO4??
?-i ? 06, P4 P4 :0 -? d ?;i

GRADING OF OSTEOGENIC SARCOMA

TABLE II.

Case.          Cell nuclei

per h.p. field.
R. D-               188

148
156
124
112
144
Mean of 6     .      145

157     .   D. J. A-
(section of irradiated

tissue)

Mean of 8

U)

C.)

cd

0

0

Cz

344
332
284
280
296
269
273
321
300

litotic
ratio.
43
39

36   1 S.D. 2-408.
41

39     Coefficient o
40         6'068 r
39- 67

f variation
per cent.

111   I

111 1

103 I

122   I S.D. 9*411.
118

134     Coefficient of variation
124   I     7 954 percent.
123

118-25

0       20     40      60     80

Age ( by decades)

FIG. 1.-Age distribution of all cases of osteogenic sarcoma in this series.

1. Age distribution.-This is shown graphically in Fig. 1. The typical two-
peaked curve shows the main incidence to be (a) in the second decade, 12 (33*3
per cent), (b) in the seventh decade, 5 (13-8 per cent).

2. Sex di8tributtion.-Males, 25 (69.5 per cent), females. 11 (30.5 per cent).

3. Site di8tribution.-(i) Lower limb, 20 (56 per cent), of which 17 (47 per cent)
were tumours, primary in the femur; (ii) upper limb, 8 (22 per cent); (iii) skull,
3 (8 per cent); (iv) axial skeleton, 5 (14 per cent).

No.
66

31,

M

I

C. H. G. PRICE

4. Incidence of Paget's disease.--This was present in 7 cases (19.5 per cent).
Of these 7 cases the mean age was 70 years, with an average duration of 9 9
months.

5. Five-year survival rate.--Six cases lived for this minimal period. As there
are also 8 cases included in the series that are still alive and when last reported
were well, but for periods less than 5 years, the 5-year survival rate is best ex-
pressed as 6 in 28 cases-i.e., 21-4 per cent.

Two of these cases died at respectively 77 and 96 months with multiple pul-
monary metastases, and the writer fully supports the view that 5 years is too
short a period on which to base estimates of rate of cure in patients with osteogenic
sarcoma.

These brief data may be compared with those published on larger series by
the following: Christensen (1925), Coley and Pool (1940), the results of the New
York Memorial Hospital series mentioned by Coley (1949), Geschickter and Cope-
land (1949), the series reported by the British Empire Cancer Campaign (1949),
and Platt (1951).

As may be expected with a relatively small group of cases, there are some
differences from the larger published series, e.g., an unduly high incidence of
femoral tumtours, but the broad agreement would suggest that this group may
be regarded as a fair sample from which certain conclusions may tentatively be
reached.

Clinical Grading of the Tumours.

The data on which the clinical assessment of degree of malignancy has been
based has been considered from the following points of view:

1. Total time of duration of the morbid process. The data shown in Table
III give the figures for each case as time in months. This has first been divided
into two sections, ante- and post-diagnosis, and then the total combined timle of
survival to the date of the last recorded examination of the patient.

2. Site of origin of the growth.

3. Age of the patient at time of onset of symptoms.
4. Complicating factors, e.g., other skeletal disease.

5. A radiological subdivision of the tumours into those which are mainly
osteolytic or osteoblastic. The histological analysis will be discussed in respect
of these factors under the appropriate sections.

In attempting to arrive at some appreciation of the clinical degree of malig-
nancy of each individual tumour for purposes of comparison, it was decided that
the best clinical guide available could be obtained from consideration of the
growth rate of each sarconma, and also the total time of survival of each case fronm
the date of presentation of the first relevant symptom. This method has certain
inherent disadvantages, e.g., a tumour in an axial bone would prove more rapidly
fatal than a similar one in the appendicular skeleton. It is also difficult to assess
the influence of treatment upon the time of survival between diagnosis and death.
Some indication of these factors is given later. In general terms the balance
of prolonged life would be aweighted heavily in favour of the patient with a tumou'r
distally situated in a limb bone, rather than the patient with a sarcoma of the
axial skeleton. However, in terms of total effect of the tumour upon the host,
the total survival time is probably the best means of measurement of relative
clinical malignancy in this type of growth.

52

GRADING OF OSTEOGENIC SARCOMA

AP4 4   P4 P4 P            ;' 4;  P . A;

4D

0

--4 ~

0

o

o  CO

0@ 10

o  +

o -

c'  CB  t-

P ,

.

-6

* i

.   .   .   .

t-  + X -+ *
t -  t - a( aq Q

L -X 0I* all

. .* . .

t -  00 C1. (M c

*. . . .

10  O0 0 =

0  0t   (   -

*^

'I'll

*Di

+oD

2

m

o4_

oD

Fi0 CD

.D

.    .   .   .   .   .      .    *

COOD 10 If.) 1    eq        COCO    C Oa

0e0'X4OCD C CO      Os    -0     0
-*  .    *     -          --*     -

* . *   .   .   .  *               -

*****4           *    .1    **4

- t- t- = 10

.  . * . .

-  44 It- 1

-   L--4

00O0 =          4'  d  P-
P-  P-      CO

. .*       .   .  .   .   .   .   .   .

-    0          - .   -

* * * .   .   .   .  .   .   .   .   .   .

0O10  CO  .4 0-4t0  O   C  0  1  C  O
_     4   _       _I m  _  _ - t  t-CI

. .*       .   .  .   .   .   .   .   .

4;P4o9:i  X':i;i: ;4

.        .   .   .  .   .   .   .   .   .

OCD  10 0 C _-4  C0C O_C *
_01 C* C o "i      t-oo oo 00C5

Ca

)      t'-   -*         CO1   1-
E-     01     01        01    tr-

1

-

IO I

COW,

4

-4
f-4
0

4    .4

4a  10
0 m

53

0- *q
p: q

r,     Ca
ii4   -F

PA
q'

.-q

- -

CD C 4  O 0
t-- _ -

'4-. a

(6

bX     <

V4     t
C      C

6

0
CO

I

:     :   ..  -.   .- P4     :   : P4    :           gi        .     :

PI;

.     .   .   .     .     .   .   .   .   .                    .     .

C. H. G. PRICE

H S.

-14

I.

0
0 1

.4
C44

0
0
-4Q

:4Z
04Q

A13

o -0 z

EH

0 ..

0

z . b

1 -0

x.  e

*- *

lit IR1-

01

14 c

0

ea m

~41

I

* I

00 CO

*~ -

t- It

co t-

Cot

01 t

?;

COm
9

o 01

I I

CO 01~

m cq

o -

3101

rr c

01 C

10 CO
01

* 10

01

5   :

CO

-0

*

C:

*4

6 d

P _~

_-4 Cq
Cq CS

. . . . . .
: ::P P4p,::

. . . . . .

CO  ~ ~ ~ O

-0(m a - -- -,
-q     N Eq   Oq   0-   1
0 14   ( 4   CB- C O

44~~~~P

Iq     I- - I

cq a  qcqa

54

.!?i
w

c12
. leb9

4Q
9
0

T
0--i

P
?4
m

9

14.

0

0

:

._

-4

0

._

S

.5

0_

0
0

4._

oCO
:4CO

0.

01._

0 *-.
24

S o

.140

II II

.0
w -

II It
p4

:4,

.2

44

0
:4

. . . .

GRADING OF OSTEOGENIC SARCOMA

On reference to Table IV, which sets out the clinical grading of the 29 osteo-
genic sarcomata of polyhedral cell type, it will be noted-

1. That the cases graded I and II are juveniles with the exception of No. 31.7,
C. P-, aged 89, and No. 191, J. E. S-, aged 38.

2. All these tumours (Grades I and II) have arisen in the appendicular
skeleton.

3. The tumours of the axial skeleton have all been graded III.

In the group of spindle-celled sarcomata listed in Table V the age difference
does not appear to apply at all, and there were no tumours of this tvrpa in the
series sited in the axial skeleton.

Histological Grading.

The tumours have been graded I, II and III in ascending order of degree of
malignancy, the annotation being largely based upon that adopted for tumours
of epithelial origin. From considerations other than histological, e.g., site,
concomitant skeletal disease, etc., it is possible also to indicate tumours which
should also be regarded as Grade III plus. The various factors to be considered
in the histological survey are compared with the clinical grading based upon the
factors mentioned above.

Cell Type and Stroma.

It is possible to divide the whole group of 36 sarcomata into two major sub-
groups according to the main cell type present. These are:

A. Polyhedral and angular-29 cases.

B. Spindle-celled or fusiform--7 cases.

A. Polyhedral-celled sarcomata.

It is impossible here to escape the conclusion that the tumour-cell is primarily
a neoplastic variant of the osteoblast. In a section of a well differentiated tumour
the cells may show the somewhat basophilic cytoplasm and well-defined vesicular
nucleus with 1 to 3 characteristic eosinophilic nucleoli seen in the normal osteo-
blast. The slight basophilia tends to diminish during mitotic activity, and the
cell cytoplasm then stains a brighter pink with dyes of the eosin type. From
observations on these tumours and other bony conditions, the writer has acquired
the view that one of the essential functions of the osteoblast is the formation
of osteoid tissue-the precursor of bone, from which under suitable conditions
of substrate the formation of bone may occur, possibly with no further part
being taken in the process by the osteoblast. Bone should be regarded as one
of the end-products of a series of complicated and balanced chemical reactions,
and modification of the several interacting factors may favour its deposition or
dissolution. This view of the function of the osteoblast is largely in accord with
that generally held, but modifies the function of the osteoblast as conceived by
Leriche and Policard (1928), who mainly attributed to this cell a reactive role.

It has been noted also, however, that the osteoblast may be phagocytic as
mentioned by Leriche and Policard (1928) quoting Dubreuil (1910), and by
Bast (1924).

It is not uncommon to see active osteoblasts " dusted " with blood pigment
derived from adjacent haemorrhage. This is not infrequently found in young

55

C. Hl. G. PRICE

CD u
4-

0

0  00

~~~  0   ~~~~~~C ~ ~ 0 2

CD~~~~~~b

"        CD

cc ~ ~ ~  ~   *

.4   -

+++~~

$w+++ +  ++  *.+t

C > b  bs>e:

ic O 5  0r-  c _c

v O w              >

(1)  .   C)    a)

g      . 0     0

0           (:) 0    0  i
P - 4       gla .4 0

. 0

0:    a        ? 4

(D            (3)

?r-      0  ?r

. 1-1.v  2,4     (D 0

C)  !z  0

0     4D    0  0      :!? 0

4", zx         04 z

H - t- . .    1-- H H H . . . . .

" "   --    ---Q...~  q1-4  .- 0- ~-

_O  -  = v 3n 10 n   - _q CZ r- = C O -_

-        _-  _- -

0tX N   -   O   C   O "~ C   ci  -C

t  - CO.:p       E- =

c l el$ CO CO

*0101000X
*m ce  to

+ +

<    o   + +++q+

OQ + + + +

0 !

,l***

0

0

I

-* * 00
4 CO  >
0 0  c 0 1

++

++

+

IIt

.   ** .   .   .   . * * . 1.!

Qpd

=    10   C   C   '010 lp *

0O =100(= a C1  10

o 00 o m  oo     0

0 1"-4   C O 01 0   C- I a

++4++++

*++++

0          -
p4 ~ ~ ~ ~ ~ ~ ~ ~ ~ ~ -

CD         4

CB~~~~~~~~C

W   W

9    G

. *--- .

0    00o  0 o  oo  C  0 lo to  o  4  0  o  to  0

*.**** .... . . . . . . . . . . . . . .   ......  .

01-000     +   o  oo

d-++++++++++ ++++   :

++ ++         ++I+++

I I  I   I  I II I
*   I   I   .-  .2   .~  .: .-   .   .  .  .~  .4   .i

P. P  l4 --  5P  ;>  ? 4 14 ? r P4 A   -- E4 4;

P-

H

10

k
0

C4-4

0

C.,
C1

0

-

0

0

1

C  O C C 10000 = c O =   Ot-

0cOCO m 4CO 01 CO

56

W-

4S

cE1

CD

01

CO

-4
-4

. -

I

~o

10

01

o

M

0

_ ._

0

> UO

m 0
04

0 0

0 0
05W s

d0 o

0 o

C)o 0
o   p4

1?
9
LI

C8
. 'IQC.)
9

14)

81

57

GRADING OF OSTEOGENIC SARCOMA

0
o      o

m

0-

0~0
CO

0 ~ 0

S         -4

0~~~~~~~4

=,  -4 --

4)  o   0  0  0 0

bo  .

IQJIr 4  0      0

0 Ca

4) 5

m  0ZII -  o 0  - ^  31
.)   0              0

0.)~~~~~~

S~~~ n  *~       t -* *

CO I-CC  00  010

00     --~~~~~~~ -  -11

,) .4<  ~   ~  ~   0  0 .' C, ..

s)  .H  OC   ~  ~4 CO  O C

O   WbL.  . I   .  0

H  4)eeO  O ns

C 2O     0

s I  I II I 1

6 O      COb X   00

0  _OOC  COe _ e

C. H. G. PRICE

callus and in Paget's disease. It is also generally accepted that the osteoblast,
both normal and neoplastic, may produce phosphatase.

From the grounds that this cell therefore is actively concerned in the laying
down of osteoid arises the essential stromal feature of this histological sub-type
of osteogenic sarcoma, i.e., the characteristic osteoid stroma, which mav also be
accompanied by considerable amounts of collagen and ill-formed cartilage.
However, the osteoid is the essential feature. This stromal material may, how-
ever, sometimes occur in considerable amount in a tumour which is predominantly
spindle-celled, but is then usually less evident than the collagenous fibrous matrix
produced by the spindle cells. Under suitable conditions the osteoid matrix
may either calcify or ossify, more often the latter, and then produce the appear-
ance of the osteoblastic or sclerotic type of tumour. It is thus possible to sub-
divide this group further into

i. Polyhedral-celled sarcom a-osteoblastic-22.
ii.         Ditto         -osteolytic -  7.

As may be expected, there is some overlapping in stromal composition between
the polyhedral and spindle-celled groups. Of the seven tumours of the latter
type in this series one could be classified as osteoblastic, the other six being
osteolytic.

As the separation into blastic and lytic forms may largely depend on general
radiological appearances, there is sometimes the difficulty of differentiating new
tumour bone from new reactive bone and from calcified material. From the
viewpoint of the histologist the essential histological structure of any neoplasmn
occurring in a bone is more characteristically shown in an area of active growth,
the radiological manifestations of which are more frequently lytic than blastic.
Hence this feature of the amount of bony matrix is not always readily appreciable
from even a large biopsy. For this reason this subdivision will not be pursued
further in the present analysis; but it should be added, however, that a markedly
bony stroma is often indicative of a tumour of relatively low malignancy, and this
point should receive due consideration by the person who may attempt to cor-
relate the histological, radiological and clinical findings in any particular case.

From inspection of Table IV the following points are evident:

1. The degree of cellularity of the tumour expressed in terms of the number of
cells per standard field for the most cellular areas does not furnish any guide to
the degree of malignancy.

2. Cellular pleomorphism is generally, but not invariably, an indication of a
tumour of high-grade malignancy.

3. The converse relationship, whilst often observ-ed, has numerous exceptions.
4. In an approximately quantitative assessment of the amount of osteoid
stroma present in these sarcomata it is seen that there is usually a rich matrix
in tumours of lower grade malignancv, and conversely a relatively smaller amount
in the high-grade group. There are noteworthy exceptions, and the specimens
showing perhaps this stromal feature in the greatest degree were those from
BTR /66, R. D-, which were relatively acellular but had a well-developed matrix.
Where this osteoid tissue is well developed it is usual for much of it eventually
to ossify. There is some evidence suggesting that nuclear activity in a cell is
accompanied by a lowered pH of the cytoplasm, possibly also producing some
change also in the extracellular substrate in which the cells lie. Swenson (1946)

GRADING OF OSTEOGENIC SARCOMA

has shown that a low pH in a haematoma is associated with bone resorption in the
region of a fracture. It appears therefore likely that the more acid tissue fluid
which may exist in the region of rapid tumour growth is one of the factors which
would influence the extent to which the osteoid stroma would be ossified. In
support of this assertion it can be pointed out that it was a nunmber of those
tumours which showed a slow growth rate as evidenced by their mitotic ratio
and clinical survival (Nos. 6, 26, 39, 43 and 203) that showed the most marked
bone formation in their sections. There was also a moderate amount of bone
present in the stromal tissues of Nos. 66, 213, 221 and 226, but generally this
feature was less evident in the more actively growing tumours. It is emphasized
that a change in pH of the substrate is only one of a number of factors concerned
in this biochemical change. It is also wise to bear in mind a fallacy that may
arise in sampling a bony tumour, as the more easily sectioned and hence less
bony areas are frequently selected for routine diagnostic examination.

Quite a nuimber of these sarcomata also show chondroid stromal areas which
may either calcify or ossify, both changes often being seen in adjacent areas
and largely governed by two factors:

1. Suitability of the substrate.

ii. Modification of the fine collagenous fibres of the chondroid material
into a form which is denser and stranded, this change apparently being
brought about by osteoblasts. In the absence of this latter change, the
cartilage responds to its environment by calcification. In several tumours
there were also patches of mucinous material, and all these stromal
variants are indicative of the wide potential biochemical ability of the
ancestral stem cell, the fibroblast. These features do not appear to have
any special significance, from the viewpoint of grading.

5. The iimean mitotic ratio of these sarcomata decreases as one passes from
clinical Grade I to Grade III, This is mluch more evident in the separation of
clinical Grade I from the tumours of Grades II and III. It may be noted here
that based upon clinical data case BTR/136, Y. M-, has been graded I. It is
not unlikely that this tumour should be re-graded II in view of the low mitotic
ratio, and suggests that the long survival of this patient was a result of the
prompt amputation which followed upon diagnosis. In this case the time period
between the presenting symptom and radical operation was only one month,
and the case may be regarded as a considerable surgical success.
B. Spindle-celled group.

These sarcomata, as compared with the polyhedral-celled group, are generally
of a more uniform cellular structure. Tumour giant cells and nmultinucleated
giant cells of distorted osteoclast type are much less frequently seen. Histo-
genetically they appear to be related to the fibroblast, which even in its neo-
plastic variant appears on occasion to be able to modify itself to becomie an
osteoblast and form some irregular osteoid stroma. This feature was evident
in 3 out of the 7 growths included in this sub-group, and in one of the tumours
(BTR/157, D. J. A-) there was produced also a sufficient amount of new bone
to warrant its classification as an osteoblastic osteogenic sarcoma.

The inultipotent ability of the fibroblast which shows itself in the occasional
appearance of some amount of osteoid, bony or chondroid stroma was recognised

59

C. H. G. PRICE

by the American Registry of Bone Sarcoma in their 1939 (revised) classification,
and they continued to regard intrinsic fibro-sarcoma as a variant of the osteo-
genic group (Ewing, 1939). Jaffe (1947), on the other hand, commenting upon
this adheres to the stricter view and classifies fibro-sarcoma as a separate entity.
From consideration of the small group of cases here reported it would seem that
this differentiation is indistinct and serves no useful purpose.

Table V gives details of these 7 cases.

This grouip is far too small to be the basis for any definite conclusions, but
the following tentative points emerge:

1. From considerations of cell morphology, stroma, and number of cells per
unit area of the most cellular parts of the tumour in the sections examined, it
is not possible to arrive at any useful histological grading.

2. On direct comparison by grades with the tumours in the polyhedral-celled
group, those of the spindle-celled type show rather less stromal material, are on
average somewhat more cellular but less pleomorphic, are mainly osteolytic, and
the average time of survival of the patient afflicted with this type of bone sarcoma
is rather longer in Grade II and about the same in Grade III.

3. The mean growth rates of these tumours graded clinically II and III in
Table V as indicated by the group mean mitotic ratio agree fairly closely with
those obtained for the comparable clinical grades of the polyhedral-celled group
of sarcomata (Table IV).

4. In the spindle-celled group there were no tumours which appeared to
merit a clinical or histological grading of I on comparison with those of the larger
polyhedral-celled series. This appears to be confirmed on considering the figures
obtained for the mitotic ratio in these growths.

.5. On the overall picture obtained by comparing the tumours of clinical
Grades II and III of each sub-group the following figures emerge:

Duration.     Mitotic ratio,  Cells/field,

mean.      mean.
Group A. Polyhedral-celled (21)  .  12-1 months +  .  129 1  .  272
Group B. Spindle-celled  (7)  .  20-9 months +  .  128 1    .   392

6. One may perhaps from this draw the conclusion that there is some evidence
to suggest that in a series of these two histological types of osteogenic sarconma
if composed of tumours of average or more than average malignancy that the
tinme of survival is rather longer in favour of the spindle-celled type. In this
small series of cases, one obvious difficulty that has arisen has been the determina-
tion of mean survival time of the sub-groups, and it is fully appreciated that the
figures shown are only minimal inasmuch as cases have been included which are
still living. Where this has been done, it has been indicated bv the presence of
a plus sign after the figure denoting survival time in months. The inclusion of
such cases was thought to be justifiable in view of the marked difference which
can be shown in the prognosis in cases given a histological grading of I when the
problem is approached purely from the histological angle. This is dealt with
in the next section.

Analysis of Mitotic Activity of Whole Group of 36 Sarcomata.

It is widely known that a feature of the degree of malignancy of any tumour
is the number of mitoses that can be observed in the nucleii of the component
cells. It is also known that sarcomata, both spontaneous and experimental, are

60

GRADING OF OSTEOGENIC SARCOMA

mainly characterised by continued increase of tumour size, rather than by
invasion of adjacent tissues. This continued growth is partly due to the multi-
plication of the nuinber of cells of the tumour. For the purposes of this analysis
it has been assumed that the mitotic ratio is an index of the growth rate of this
series of sarcomata. Table III gives details of age, site, type of tumour, mitotic
ratio and total duration of survival for the whole group.

From this table 9O cases can be selected in which the period of time between
the appearance of the initial symptom and diagnosis is greater than 6 months.
These are cases Nos. 26, 39, 81, 96, 102, 2"03, 317, 337 and 338, and all the patients
had tumours of the appendicular skeleton. On reference to Table III again it
will be seen that 7 of the 9 tuiyours showed a mitotic ratio considerably greater
than the average for their appropriate clinical grade. Mean figures for this
group of nine cases are given:

Total months    Months duration   Mitotic ratio.

survival,     ante-diagnosis.

Mean of 9     .    40 9+      .      128       .      456

Converselv one may likewise select the type of case showing the other extreme,
i.e., there were 11 cases in which the time period between onset of symptoms
and diagnosis was one month or less. Mean figures for this group are given
below. (Cases Nos. 10, 61, 66, 70, 83, 136, 145, 163, 213, 221, 250.)

Total months    Months duraticn   Mitotic ratio.

survival.     ante-diagnosis.

Mean ofl1     .    18.3+      .     Under1     .       122

There is obviously some overlapping of individual figures between the two
groups, and 2 tumours of the axial skeleton are included in the latter larger
number.

Subject to individual differences in tolerance of patients and speed of diagnostic
methods, one may assume that the more rapidly growing tunmour will lead to the
earlier synmptomatology in tumours of a similar primary site. That being so,
the difference in the degree of mitotic activitv of the two groups of tumours is
striking, as also is the difference in the mean time of survival of the former group
as compared with the latter; and whilst 5 of the formier group are still living,
there is only one survivor in the latter group. This degree of agreement between
the mitotic ratio, ante-diagnostic period and total time of survival helps to render
more evident the fallacy in the conclusion reached by Ferguson (1940), and
supports the more reasonable explanation given by Colev (1949), that tne more
slowly growing tumour produces the less alarming symptoms.  It would seem
niore rational to associate urgency of treatment with tumours of more rapid
growth, i.e., Grades II and III, in which grades of malignancy the survival rates
will be minimal, by whatever lneans the cases are at present treated. Per contra,
in tumours of Grade I malignancv, occasional cures ma-y be expected by radio-
therapy only, e.g., No. 26, N. G-, and it is strongly suggested that the aoverning
factor in the survival of any case of osteogenic sarcoma is the combination of
site of origin and histological grading of the tumour.

Table VI shows the 36 cases of osteogenic sarcoma arranged in ascending
order according to the mitotic ratio, i.e., in order of diminishing mitotic activity.
This has been subdivided into three purely histological grades of malignancy,

61

62                                  C. H. G. PRICE

into those cases with a mitotic ratio less than 100:1, from 100:1 to 400:1, and
greater than 400:1. This may be taken as approximnating to those of greater,
average and lesser degrees of nuclear activity. Reference back to Tables IV and
V will show that all the cases there clinically graded as III appear again as Grade
III (histological) plus one case, BTR/157, D. J. A-, which was clinically graded II.

TABLE VI.-Whole Group of 36 Osteogenic Sarcomata. Histological Grading.

Months     Mitotic     Cells per  Histological Clinical grade
No.          Case.        total       ratio.    h.p. field.   grade.  Tables IVandV

66     .  R.D         .    11    .    43     .    188    .    III     .   III
273        T. H-             9    .    46     .    228    .    III     .   III

94     .  E. M.J      .     5    .    48     .    300    .    III     .   III
83        G. J-             9    .    65     .    212    .    III     .   III
212        R.M         .     7    .    65     .    196    .    III     .   III
157        D. J. A-         42+   .    73     .    356    .    III     .    II*
250        M. G-            18         83     .    284    .    III     .   III
163        D.R         .     5    .    88     .    400    .    III     .   III
155    .   F. A. G-         11         91     .    232    .    III     .   III
230        C. H-             4    .    91     .    312    .    III     .   III
106    .   J. W-             4    .    92     .    448    .    III     .   III

(S.D. 10-9)

* BTR/157, D. J. A-: Probably favourable result of treatment has led ta unduly low clinical
grading; tumour in lower end of femur.

Group based on range of mitotic ratio of less than 100.

Mean survival period of grade III (11) - 114 + months. One still living (9- 1 per cent.).

145    .   K. C.W      .    15    .    108     .    244    .    II    .     II

136    .   Y.M         .    77    .    113     .    432    .    II    .     I(?)
191    .   J. E.S      .    31+   .    125     .    220    .    I           II
213     .  E.C         .    25+   .    125     .    360    .    II     .    II
102    .   F. A. H-    .    11    .    132     .   480     .    II    .    III
337     .  J. A.B      .    17+   .    134     .    808    .    II     .    II
221     .  M. C.Ca     .     6    .    135     .    160    .    II         III
143    .   J.H         .    15    .    153     .    340    .    II    .     II
226     .  A. R.O      .     3    .    168     .    224    .    II     .   III
198    .   W. G-       .     6    .    174     .    265    .    II     .   III
299     .  F. H.B-     .    11+   .    180     .    288    .    II     .   III

70    .   A. L-       .     7    .    180     .    320    .    II     .   III
338     .  A.D         .    14+   .    183     .    344    .    II     .    II

10    .   P. C-       .     8    .    190     .    268    .    II    .    III
81    .   R. K-       .    27    .    195     .    168    .    II     .    II
169    .   M. S. A-    .    12    .    198     .    324    .    II    .    III

61    .   J. S.C      .    20    .    206     .    240    .    II     .    II
96     .  F. C.T      .    48    .    232     .    252    .    II    .     II

(S.D. 18.52)

Group based on mitotic ratio range of 100 to 400.

Mean survival of Grade II (18) - 19 d 6 + months. Five still living (27.7 per cent.).

43     .  D. C-       .    98+.       421     .    168    .     I     .    I

6    .   K. S-       .   117+.       450     .    -      .    I     .     I
26     .  N. G-            75+.       677     .    340    .    I     .     I
203     .  S. C-            36+   .    727     .    344    .     I     .    I

35     .  N.J         .    82+.       773     .    132    .    I     .     I
39     .  S. H-       .    96    .    888     .    132    .    I     .     I
317     .  C.P-        .    22+   .    926     .    264    .     I    .     I

(S.D. 34 48)

Group based on range of mitotic ratio greater than 400.

Mean survival of Grade I (7) - 75v 1 + months. Six still living (85. 6 per cent.).

The correlation coefficient for the whole series has been calculated for the two variates months
of survival and mitotic ratio. The value obtained for r was 0 6156.

GRADINIG OF OSTEOGENIC SARCOMA

Histological Grade II in Table VI includes all those cases formerlv given a
clinical grading of II, plus one case BTR/136, Y. M--, formerly given a clinical
grading of I and 8 others (Nos. 10, 70, 102, 169, 198, 221, 226, 299) which had
been clinically graded III.

The 7 cases graded I (histological) in Table VI have in each instance been
given a clinical grading of the same value.

It is evident then that on this basis any histological sub-division into Grades
II and III is indistinct, and not always in accord with the clinical interpretation
of such cases.

In the misfit in Grade III (histological) of Table VI (BTR/157, D. J. A-)
the possible explanation can again be offered that the clinical grading based
upon the consideration of survival time and other factors has been influenced by
the treatment given to the patient.

Table VII shows the cases listed in Table VI as Grade II (histological) divided
into those which appear as Grades II and III (clinical) in Tables IV and V. These
two groups have been respectively designated Groups X and Y, and on com-
parison of these the following points can be seen:

Group X (Grade II histological, Grade III clinical).

1. The average age in this group is markedly higher than in Group Y, 51- 6
years as compared with 28 9 years.

2. Excluding case BTR/299, F. H. B-, who is still alive and may eventually
qualify for a more favourable clinical grading, the cases are with one exception
all tumours of the axial skeleton, or of the upper end of the proximal long bone
of a limb.

3. In the case of BTR/226, A. R. 0-, the sarcoma at autopsy was of small
size, but had produced collapse of a dorsal vertebra with marked angulation of
the spine and compression of the cord. This may be compared with BTR/143,
J. H--, in which a vertebral sarcoma occupied a more dorsi-lateral position and
produced a very much larger soft tissue mass beneath the erector spinae muscles.
These observations emphasize the bearing of the factor of site uipon the clinical
interpretation of any attempt to grade these tumours histologically.

4. Three cases of Group X (Nos. 70, 198 and 299) were complicated by well-
marked Paget's disease, and there is some suspicion that sub-clinical Paget's
disease may have been present also in case BTR/169, M. S. A-, in which the
tumour had its origin in the ilium. No cases of Group Y showed any evidence
of this skeletal complication.

5. After evaluation therefore of the mitotic ratio and allocation of the tumour
to the appropriate histological grade, it iB suggested that a proximal site, more
particularly in the axial skeleton, advanced age or concomitant Paget's disease
(these latter two features may possibly refer to the same aggravating feature),
would indicate that the grading should be advanced to at least the next malig-
nant grade, i.e., I to II, II to III and III to III+ when the case is considered
in toto. Per contra it should be appreciated that a distal site, juvenility and a
relatively low M.R. may be associated with a period of survival of 15 to 24 months.
This is shown by case BTR/250, M. G-, aged 14, mitotic ratio 83: 1, sarcoma of
lower end of femur, who survived for a total period of 18 months. Another case
supporting this view was BTR/157, D. J. A-, aged 27, mitotic ratio 73: 1,
sarcoma of lower end of femur, who was alive and symptom-free 42 months from

63

C. H. G. PRICE

a:

0q

Cf?

0

0 U o o
4-     ^ X  n

0          0 00e  s
-,.     ? ~ -.    4.o  o

a:      t         et
->      d

4-Z    +

00

s~~ ~~~   c

6E

bE   O 44@ ~ 0

+

0

C)

I  - -' 0  E,

01 CO 0.1P

bD

. .

.--

-4
* -

2

.    .   .   .   .   .

+
I    4L  0 m   l d

0

C.)

"0

^ ^    4p >. 4 4 >,

00 00O1-0

p 0 OM

P-

o     .-

0..5 =C

Lo     V-  aq C

Ct C = 0 C) all 00 x

,-C M     =   c0 0 C -4  c

.

1.

* -

*    II

0 l

cq   >

0I

-; *; cs ce c: r ce X 0 01'0m~ 0  _  o  cq o u O x   0. 1  O  *

. -   -- -  - -  -- - - - 0 in   a :

........   ~~......  . .

* - a

0~~~~

00~~~~~~~

0                4)

o  111  1 1  1  i   AI  I  I  I 1  ?  0

6 ?vQ I;1       vxl?= 0v'

a: ?  ? X      Me e ? h .c I I II Q

0  0 1 0 1 -   =   0 0  ( .  CO )

I   0101 q   -  cli  -4

CO c' r  O  -  c t -

"d 0- CO m C" X 0 CO

_    1  C O   -  C O C

64

a.,
0%

pi

~-

za

c3

d3
li
.5
u

F-

.- )

*- .-

Ca

00

, o-
*eQ

o

Ct
00e

c3

0

* H

C4

GRADING OF OSTEOGENIC SARCOMA

the date of the first symptom. However, even with this small group of cases it
is evident that it is unusual for a long period of survival to be associated with a
mitotic ratio of the tumour falling within the maximum observed range of 43:1 to
92:1. This feature can be well appreciated from Fig. 2, which depicts the per
cent survival by years of the cases in the three histological grades of Table VI.
Histological Grade I of Table VI.

It would appear from the tabular data that it is possible to distinguish
histologically in a series of cases of osteogenic sarcoma those tumours which may
be graded I (histological). The mean mitotic ratio for the whole group of 36
sarcomata was 238:1 cell nuclei. If one selects as a separate group (P) the 7 cases
in which the mitotic ratio is markedly greater than the average the following
figures are obtained (No. 6, 26, 35, 39, 43, 203, 317):

Total.     Months survival.  5-year survival.   Mean M.R.
Mean of 7          75-1+      .  5 (71 per cent.)  .  695: 1
Mean of 29         14-1+      .  1 (3 4 per cent.)  .  128: 1

I'

to)
0

L._

uz

L.

0        1       2       3       4       5       6       7

Time (years)

FIG. 2.-Percentage survival of Grades I, II and III (histological) shown at yearly intervals.

x      x Histological Grade I (7). Mean survival 75 1 months.

*       0 Histological Grade II (18). Mean survival 15 * 8 months.

* -         Histological Grade III (11). Mean survival 11 - 4 months.

The marked difference in the prognosis in these separated groups of cases can
again be well seen from Fig. 2. It would be of the greatest value to subject many
further cases to this type of analysis, to confirm if possible the significance of the
mitotic ratio and to find its mean value based upon the examination of a much
larger series of tumours. The number of sarcomata found in this present series
of 36 tumours in which the mitotic ratio is appreciably greater than the mean
figure is 19-4 per cent of the total. It will be noted that this figure is approxi-
mately of the same order as the 5-year survival rate (21-4 per cent). Reference

5

65

I
I

C. H. G. PRICE

to Table I shows the treatment which was given to these 7 cases. As can be
seen, surgical success may occur in the treatment of cases of higher-grade malig-
nancy (II and III), but such is unusual. The analysis presented above suggests
that calculation of the mitotic ratio at the time of diagnosis by the pathologist
will enable him to indicate the grade of his histological malignancy, and hence
some idea of the prognosis may be determinable for any particular case with
more certainty. If this method can be firmly established it should be of great
assistance in the difficult decision over upper limb amputations. There are 3
such cases in this series (No. 26, 203 and 317), 2 of which have been treated by
radiotherapy only, and one by radiotherapy followed after 18 months by dia-
physectomy on account of renewed and progressive pain. In cases No. 26 and
203 a good prognosis can be given with reasonable confidence; this, however,
is rather more guarded in case No. 317 in view of the patient's advanced age
and concomitant Paget's disease, although in this latter case the site in the lower
end of the radius of the tumour does not suggest a rapidly fatal issue.

In view of the figures given for the mitotic ratio in this group of sarcomata,
it is interesting to compare these with figures obtained for similar investigations
on non-neoplastic specimens. (Table VIII).

TABLE VIII.-Mitotic Ratios in Non-neoplastic Specimens.

No.       Initials.      Condition.         M.R.           M.R.

osteoblasts.   fibroblasts.
343   .   M. G. C-  .      Callus    .    >5000: 1   .    >5000: 1
370   .   H. J. 0-  . Myositis ossificans .  >2500: 1  .  >2500: 1
371   .   G. F. M-  .  Osteochondroma .    3300: 1   .    >2500: 1
341   .   0. J. N-  .     Osteoma    .     1250: 1   .    >2500: 1
352   .   M. E-     . Fibrous dysplasia  .  >2500: 1  .   >2500: 1
339   .   B. A-     .    ,,    ,,    .    >2500: 1   .    >2500: 1

In each of these specimens of tissue there was marked cellular functional
activity with the formation of much new osteoid and bone. The mitotic ratio
in all these specimens is of a different order from those found in the general run
of the group of osteogenic sarcomata. Apart from the histological features,
appreciation of this fact may be of considerable assistance when attempting to
reach a decision on an atypical section of bony material. That the very slightly
greater degree of mitotic activity was found here related to the osteoblast, and
not the fibroblast, if confirmable, will strengthen the concept of the histogenesis
of the polyhedral-celled type of osteogenic sarcoma, and support the assertion
of Geschickter and Copeland (1949) that such tumours are derived from osteo-
blasts.

CONCLUSIONS.

1. The series of cases of osteogenic sarcoma here reported, although small in
number, appears to conform with the main features of much larger groups
formerly published.

2. By the application of histological methods only, it is possible to grade
these sarcomata I, II and III in ascending order of malignancy, the scheme
adopted being applicable to tumours of either predominantly a polyhedral or
spindle-celled sub-type.

3. The amount and type of tumour matrix, whilst of some indicative value,
are not reliable criteria for assessing the histological grade of these growths.

66

GRADING OF OSTEOGENIC SARCOMA

4. The degree of cellularity and morphology of the component cells of the
tumours are features of some value to be considered in grading osteogenic
sarcomata, but the main factor to be observed is the degree of nuclear activity
which may be estimated by the mitotic ratio.

5. The mean mitotic ratio for this series is 238 resting nuclei to one nucleus
in mitosis. Analysis of the group gives the following ranges:

Grade I:   M.R. greater than 400 to 1.

Grade II:  M.R. from 400 to 1 down to 100 to 1.
Grade III: M.R. less than 100 to 1.

6. It appears umusual to find a short total survival period of any case associated
with a M.R. greater than 400 to 1. The converse may be encountered, and is
probably an index of other features of the tumour, e.g., site, patient's age, etc.,
or of the effects of treatment.

7. It is suggested that the site of the tumour and its histological grading are
the factors bearing most significantly upon the prognosis, and it has been found
that the five-year survival rate is almost identical with the incidence of tumours
of histological Grade I.

8. The mean mitotic ratio for this group of sarcomata is markedly different
from that which has been found in the examination of presumably related cells
in non-neoplastic conditions.

The author wishes to record his great indebtedness to the late Dr. S. Bryan
Adams, sometime Director of the Radiotherapy Department of the Bristol Royal
Hospital, through whose energy and initiative the Bristol Bone Tumour Register
came into being. He was also directly and personally concerned in the treat-
ment of many of the earlier cases, and the collection of their clinical and radio-
logical records. Much thanks are also justly due to the members of the Com-
mittee of the Register for their co-operation in the collection and discussion of
all the cases included in this series. Material has been derived from the source-
indicated below, and to the surgeons, radiotherapists, radiologists and path-
ologists who have referred cases to the Register, due recognition and thanks
for their assistance are here rendered:

1. Radiotherapy Department and Department of Surgery, Bristol
Royal Hospital. Cases No. 6, 10, 26, 35, 39, 61, 66, 70, 81, 83, 94, 96,
102, 145, 155, 157, 169, 203, 212, 213, 273, 317, 338.

2. A. L. Eyre-Brook, Esq., F.R.C.S. Cases No. 43, 163, 198, 230.

3. Dr. R. C. Hadden. Radiotherapy Department, Royal Devon and
Exeter Hospital. Cases No. 136, 250.

4. J. Bastow, Esq., F.R.C.S. Royal United Hospital, Bath. Cases
No. 143, 221, 299.

5. J. F. H. Stallman, Esq., F.R.C.S. Gloucestershire Royal Hospital.
Case No. 191.

6. Dr. 0. C. Lloyd. Department of Pathology, University of Bristol.
Cases No. 106, 226.

7. G. J. Lillie, Esq., F.R.C.S. Mount Gold Hospital, Plymouth.
Case No. 337.

67

68                            C. H. G. PRICE

The expenses of the activities of the Bristol Bone Tumour Register are defrayed
from a British Empire Cancer Campaign grant, for which invaluable support
the author's thanks are also given.

REFERENCES.
BAST, T. H.-(1924) Anat. Rec., 28, 91.

British Empire Cancer Campaign.-(1949) Ann. Rep. Brit. Emp. Cancer Campgn., 27,

323.

BRODERS, A. C., HARGRAVE, R., AND MEYERDING, H. W.-(1939) Surg. Gynaec. Obstet.,

69, 267.

CHRISTENSEN, F. C.-(1925) Ann. Surg., 81, 1074.

COLEY, B. L.-(1949) 'Neoplasms of Bone.' New York (Hoeber), p. 268.
Idem AND POOL, J. L.-(1940) Ann. Surg., 112, 1114.

DUBREUIL, G.-(1910) C. R. Soc. Biol., Paris, 69, 189. (Quoted by Leriche and Policard

(1928).)

EWING, J.-(1939) Surg. Gynaec. Obstet., 68, 971.

FERGUSON, A. B.-(1940) J. Bone Jt. Surg., 22, 92.
GESCHICHTER, C. F.-(1932) Arch. Surg. 24. 231.

Idem AND COPELAND, M. M.-(1949) 'Tumours of Bone.' London (Lippincott), pp.

181 et seq.

GRYNFELTT, E.-(1929) Bull. Ass. fran~. Cancer, 18, 360.
JAFFE, H.-(1947) Bull. N.Y. Acad. Med., 23, 497.

LERICHE, R., AND POLICARD, A.-(1928) 'The Normal and Pathological Physiology

of Bone.' London (Kimpton).

PLATT, H.-(1951) Ann. Roy. Coll. Surg. Engl., 8, 87.
PREVO, S. B.-(1950) J. Bone Jt. Surg., 32A, 298.

QUICK, D., AND CUTLER, M.-(1927) Ann. Surg., 86, 810.
SIMMONS, C. C.-(1939) Surg. Gaynaec. Obstet., 68, 67.
SWENSON, O.-(1946) J. Bone Jt. Surg., 28, 288.

WILLIS, R. A.-(1948) 'Pathology of Tumours.' London (Butterworth), p. 677.

				


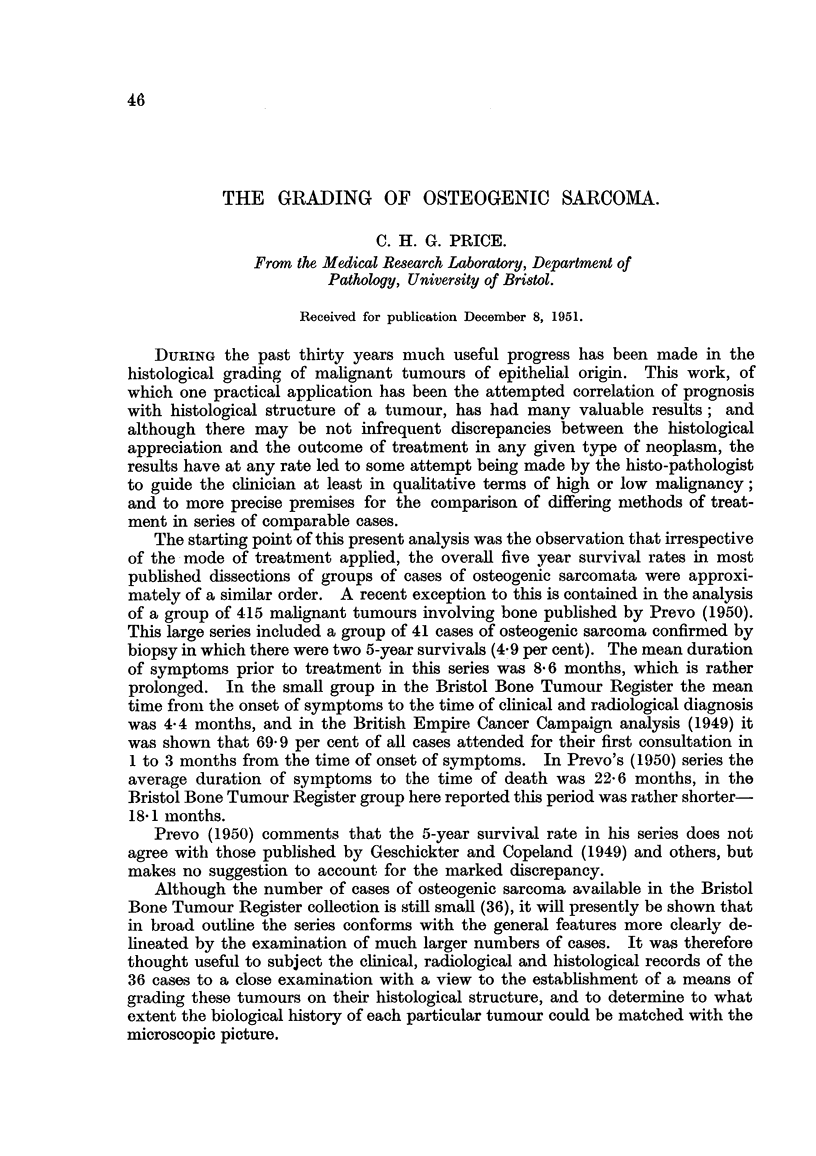

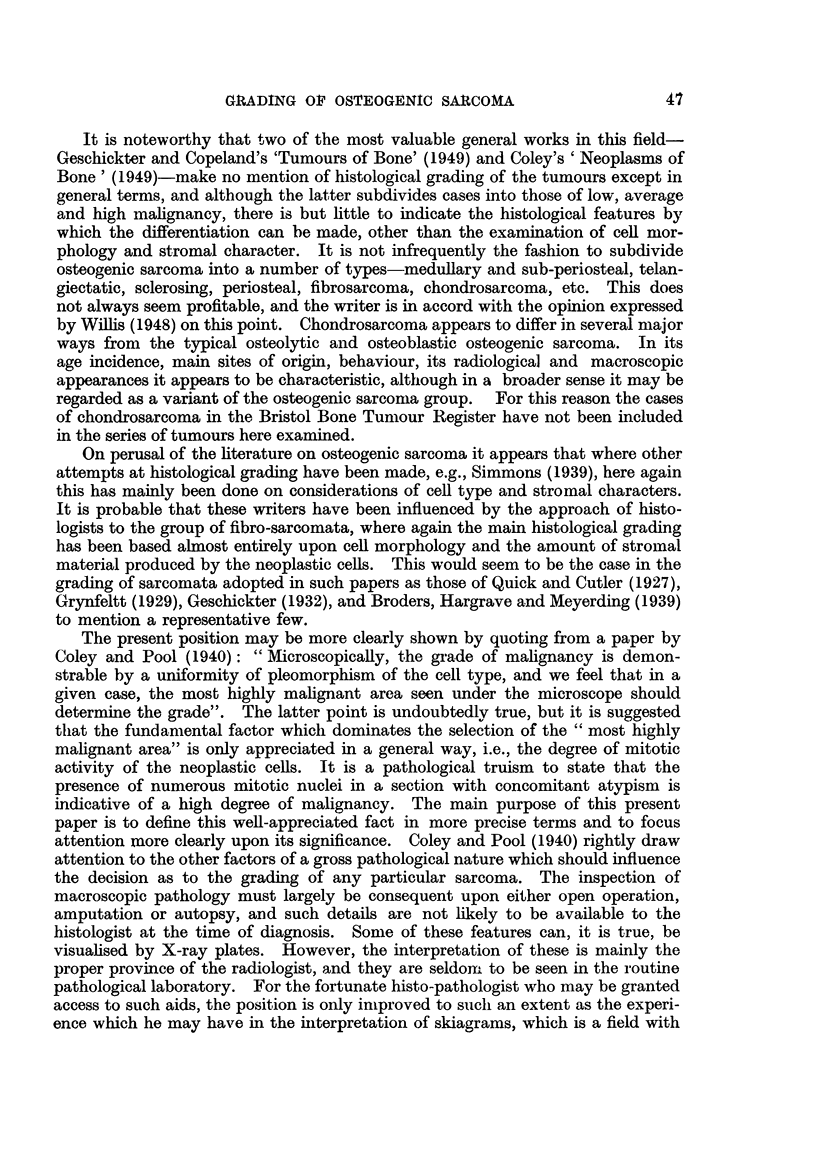

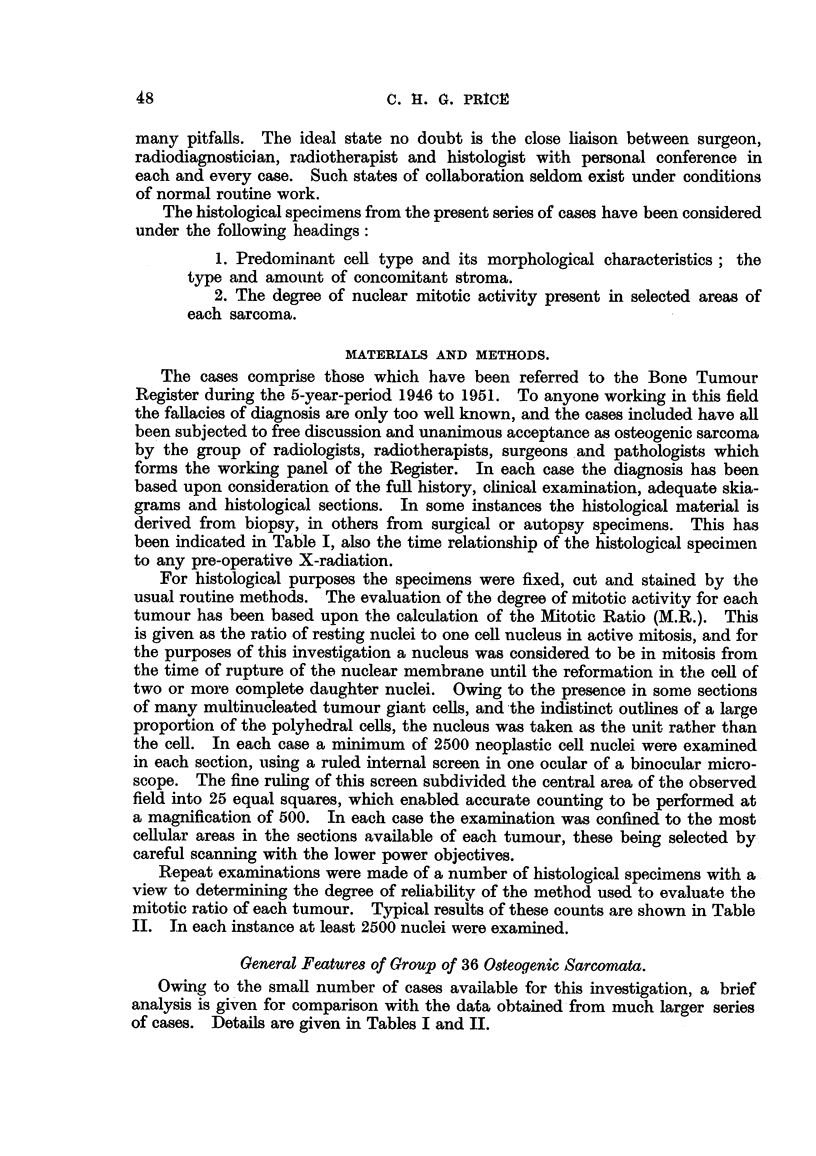

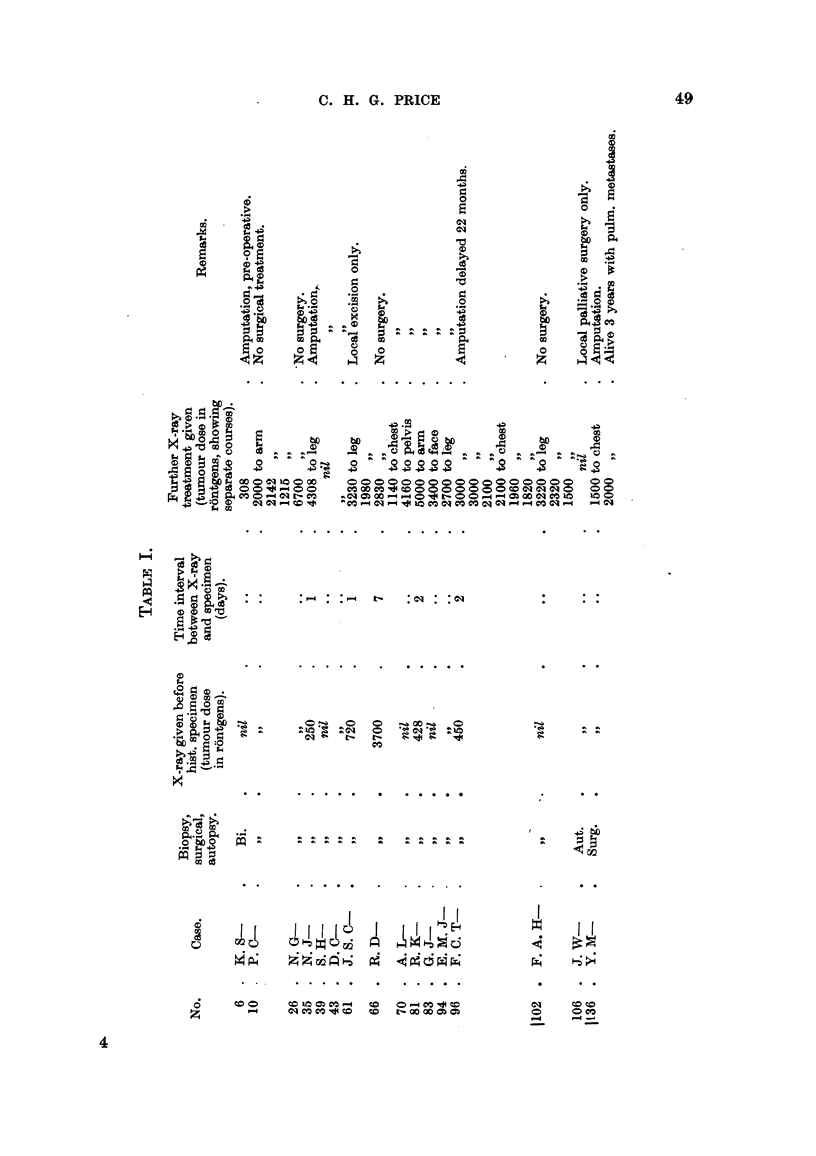

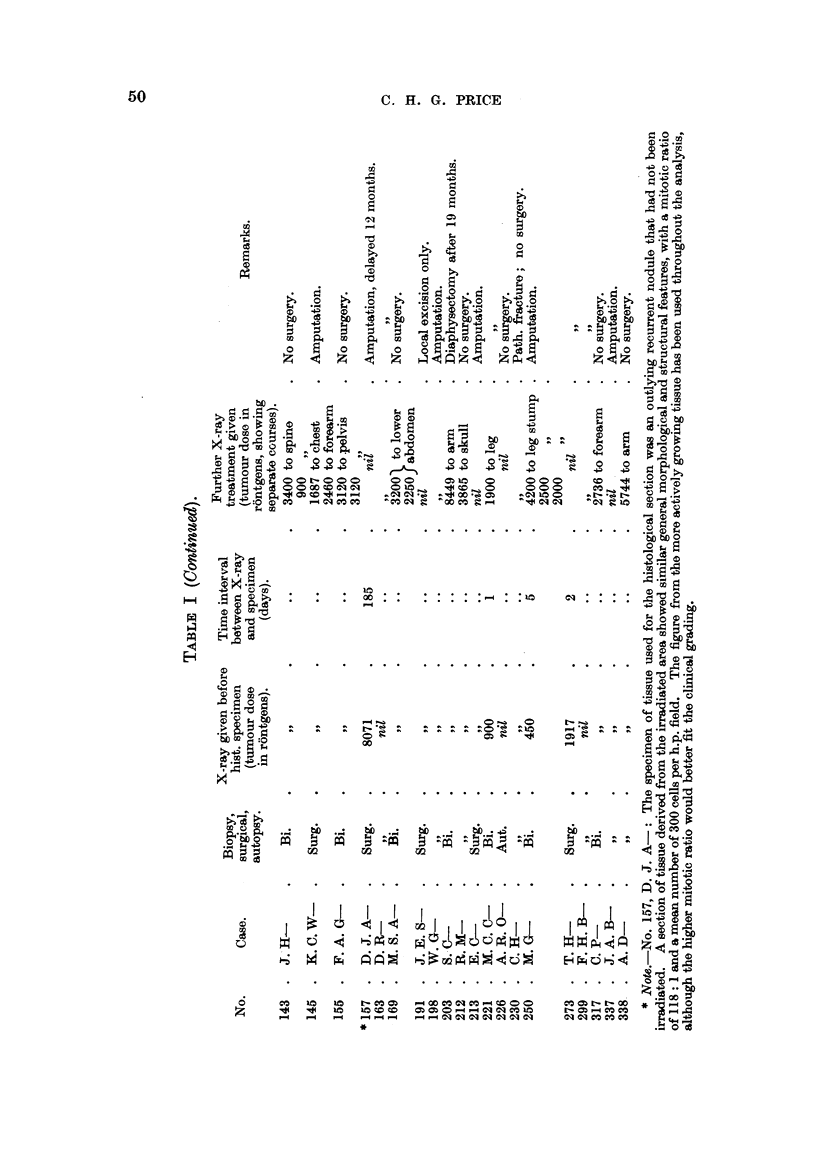

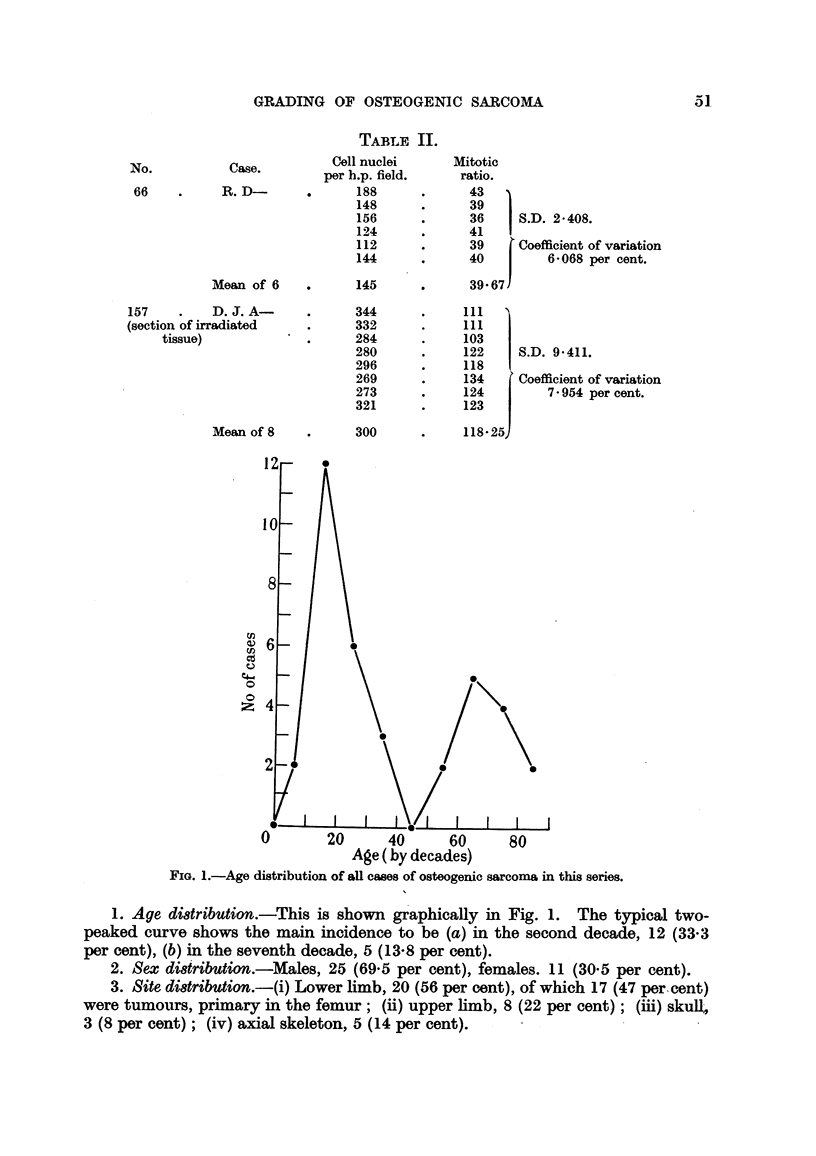

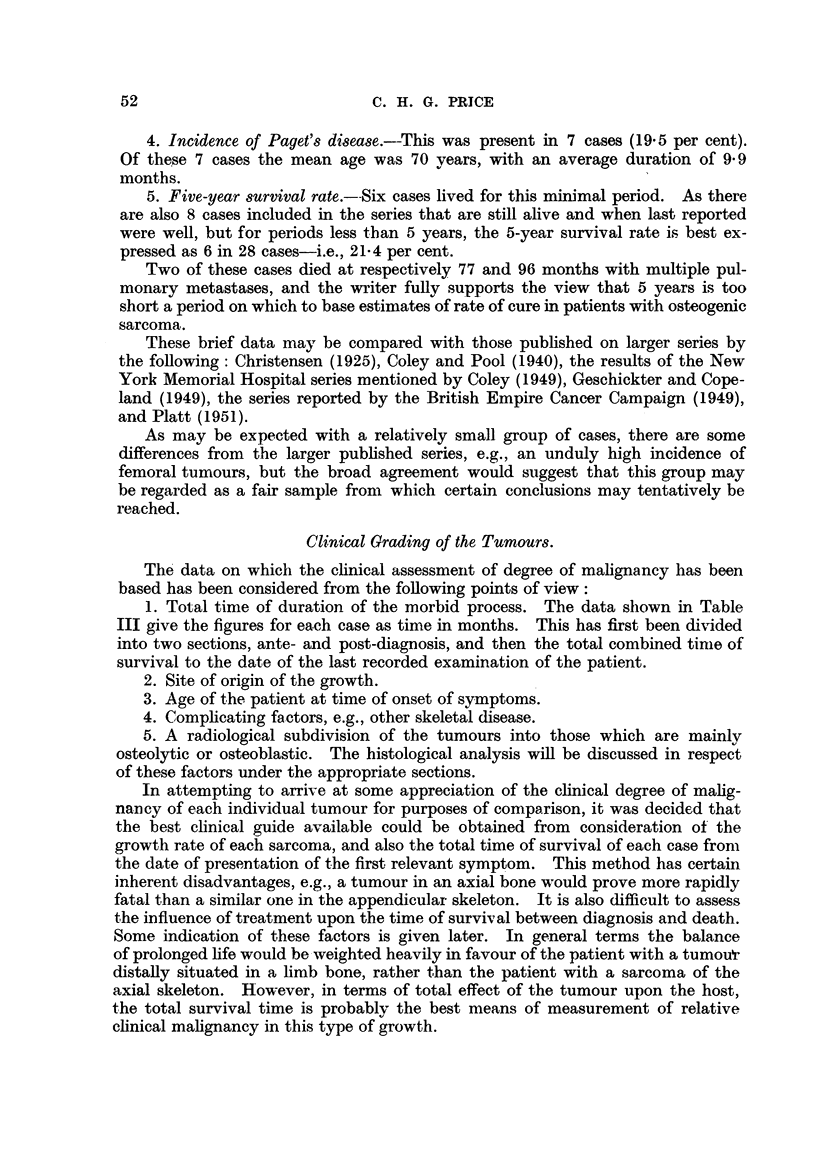

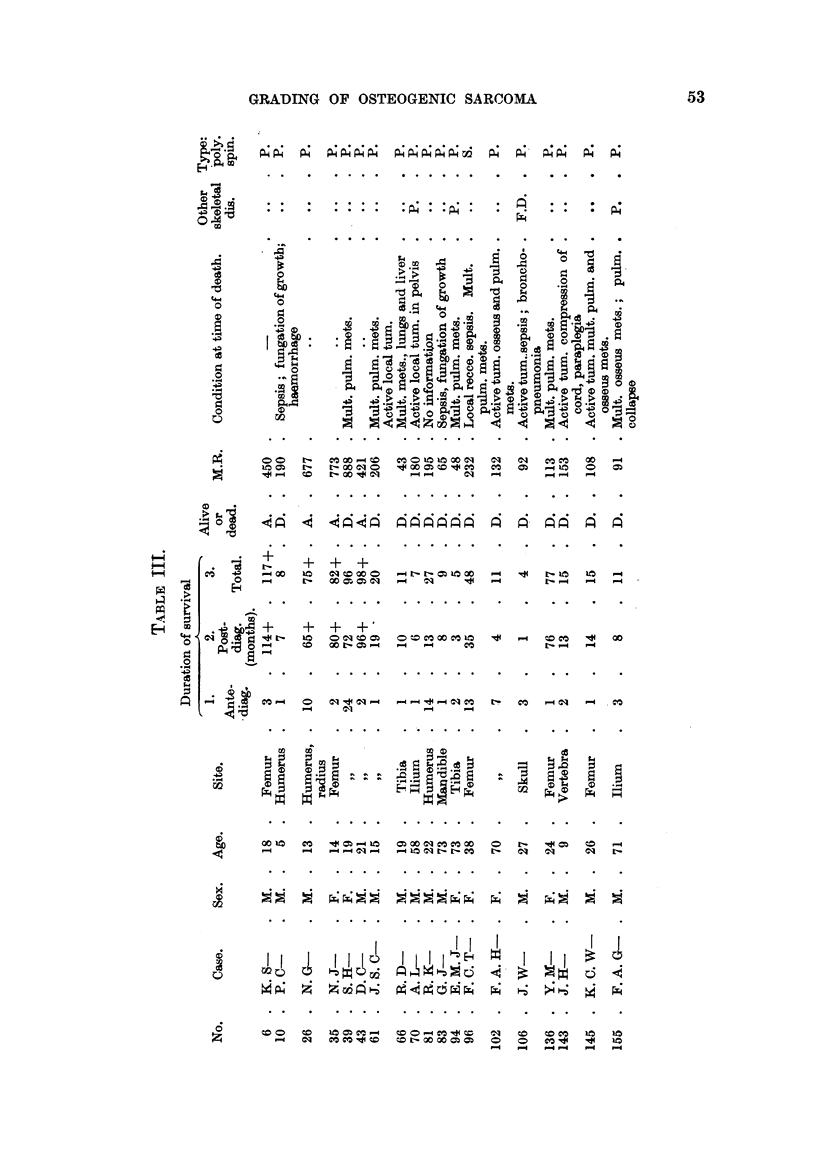

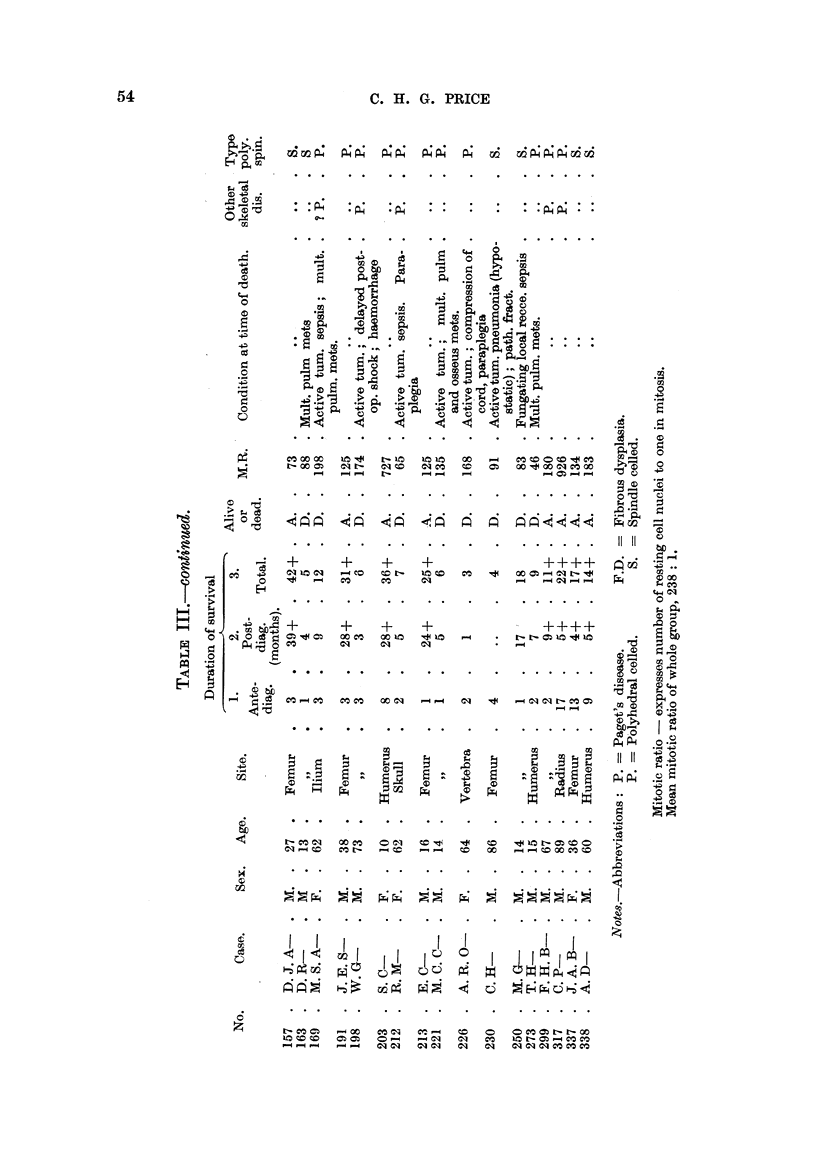

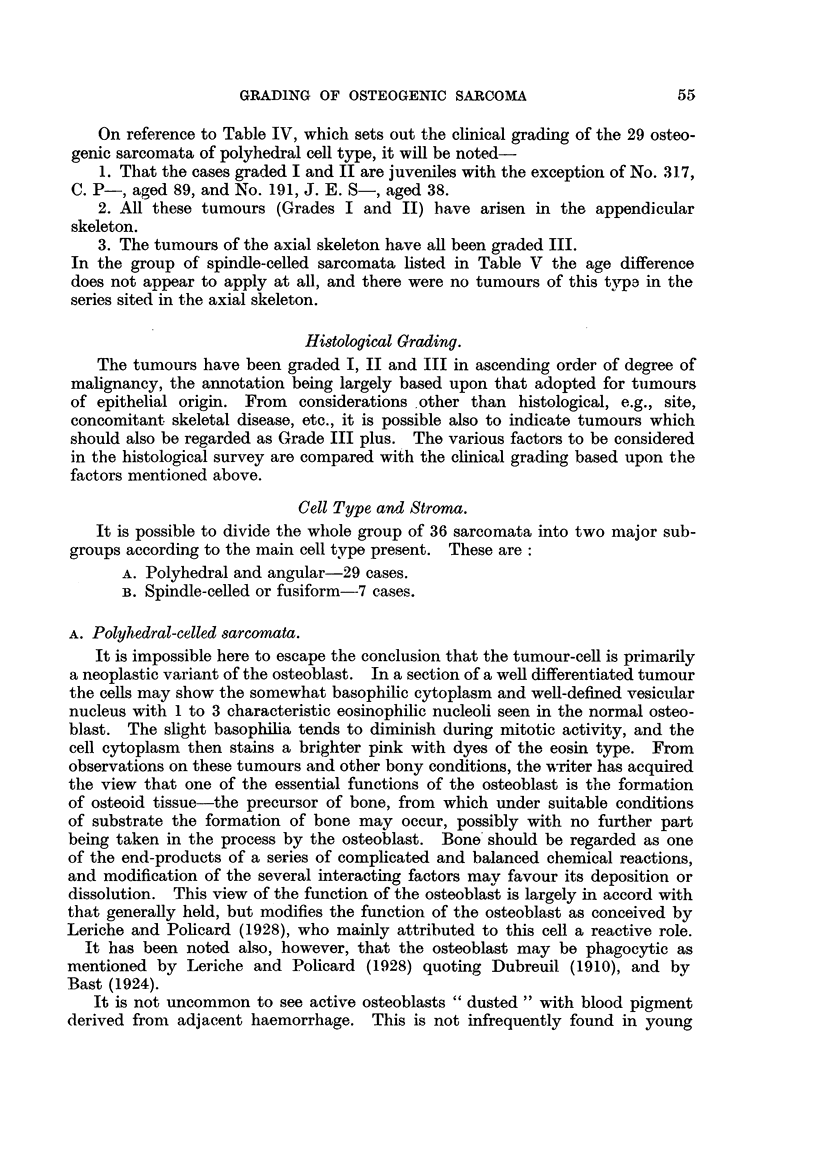

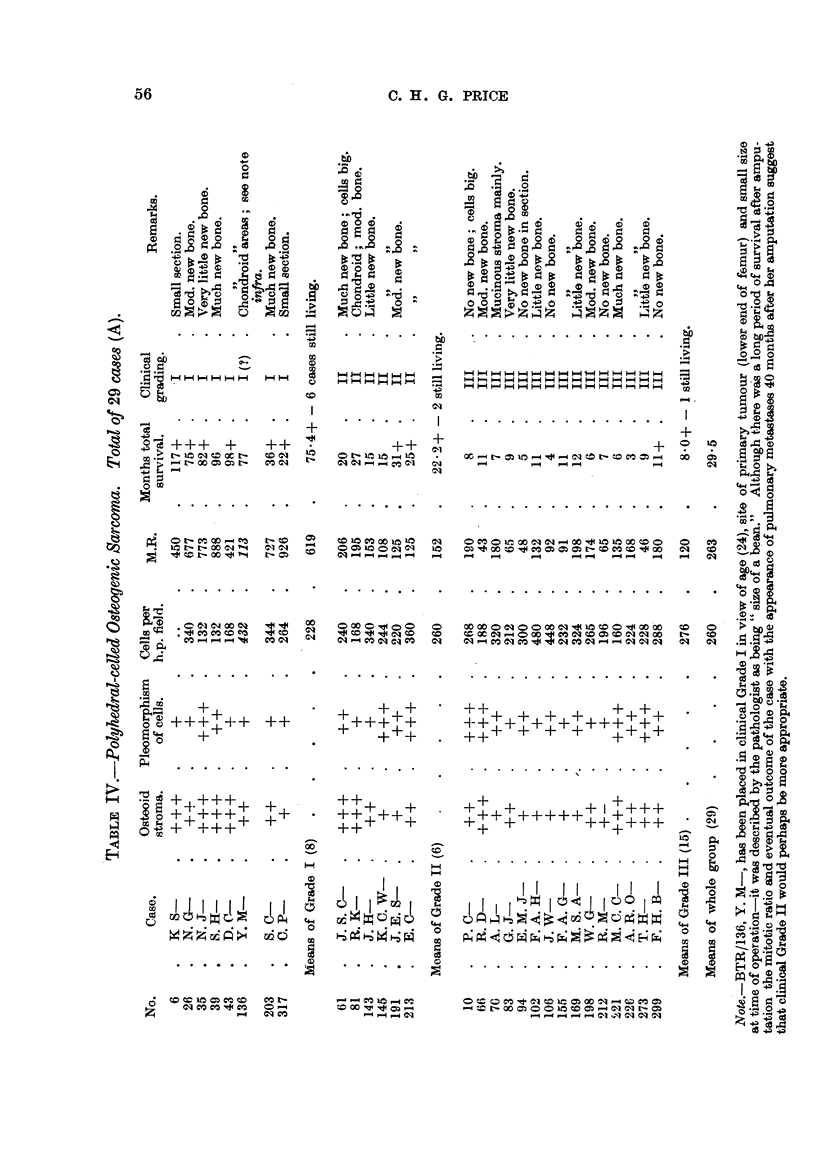

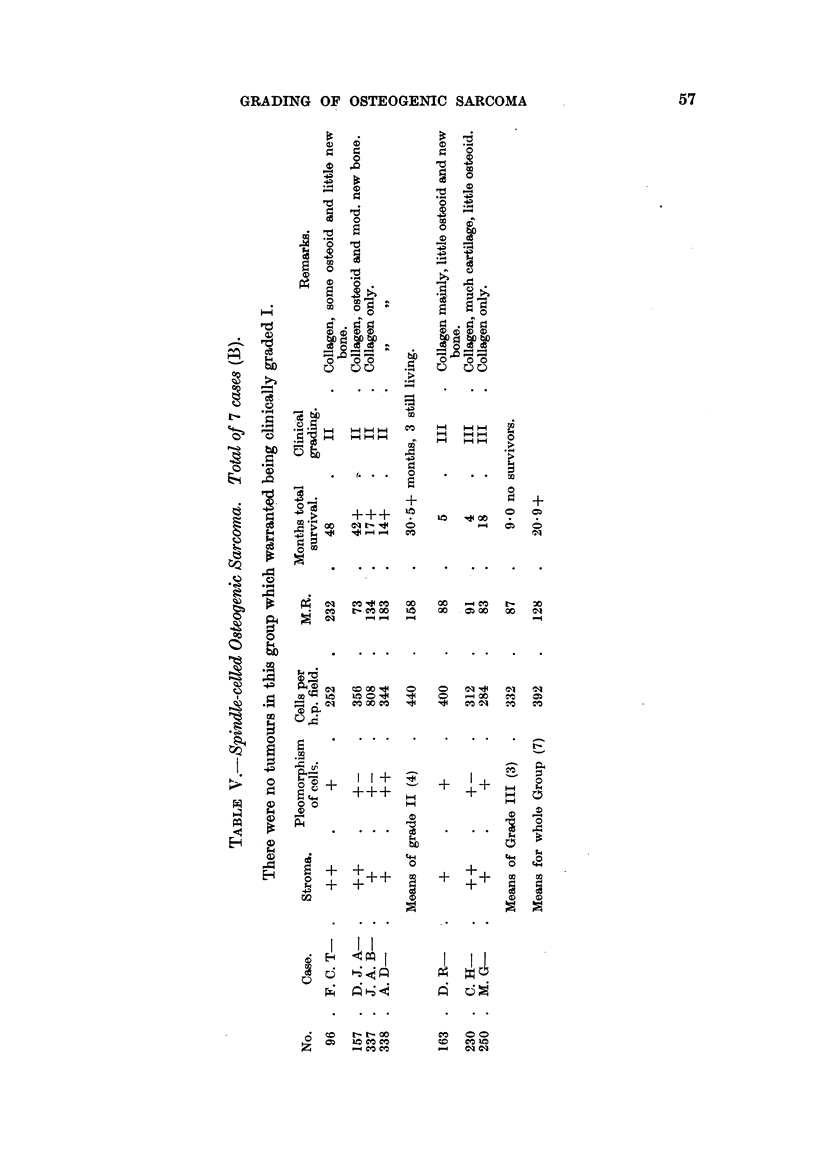

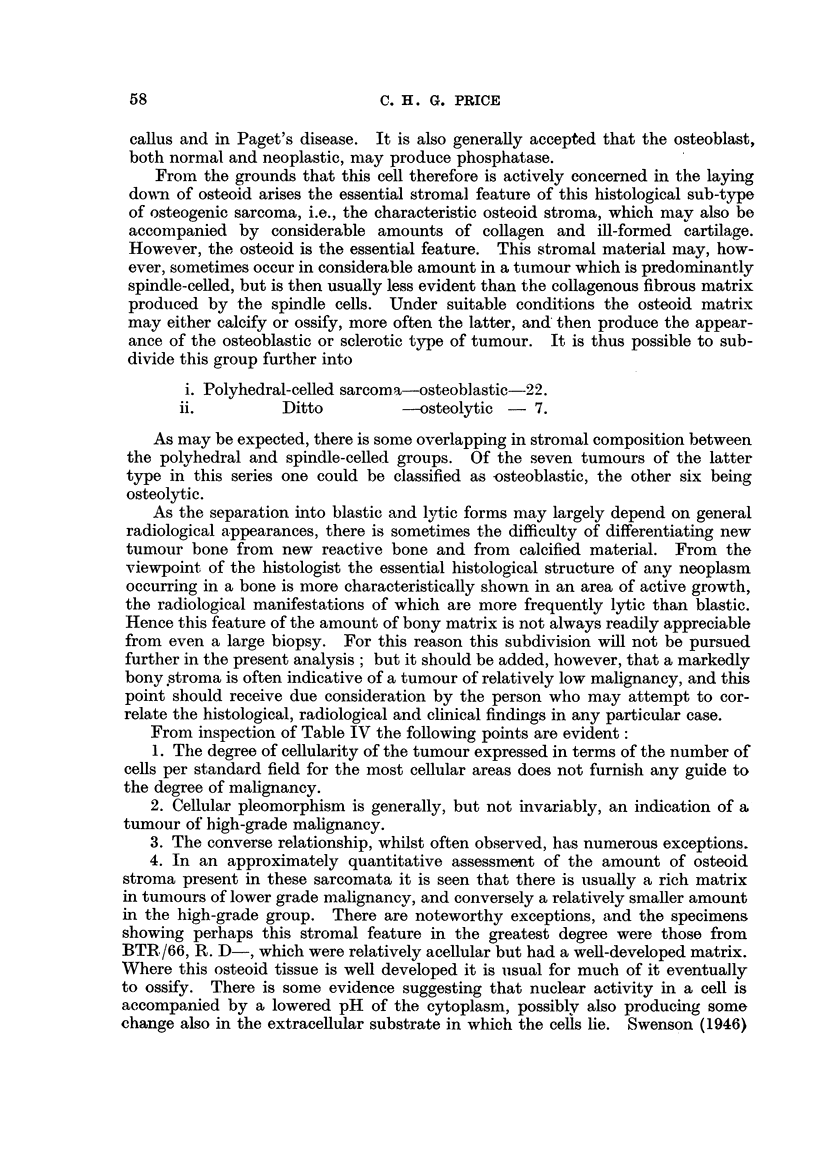

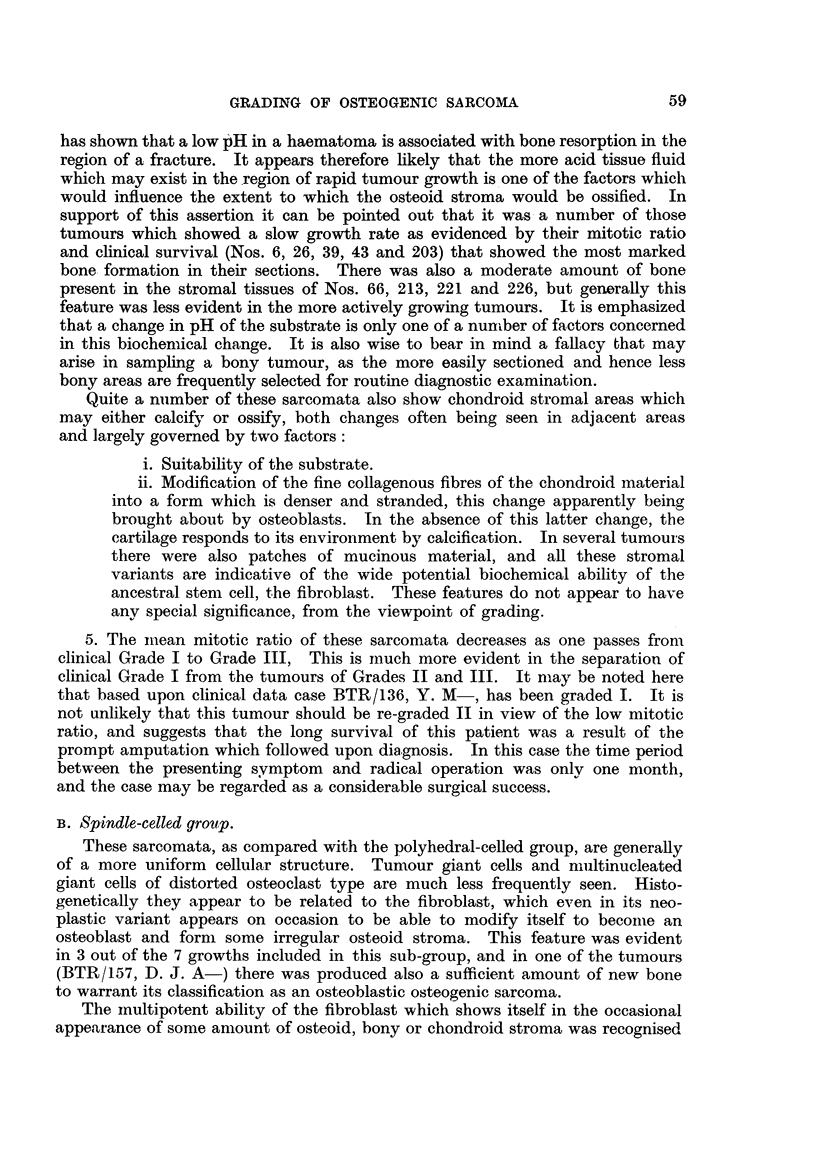

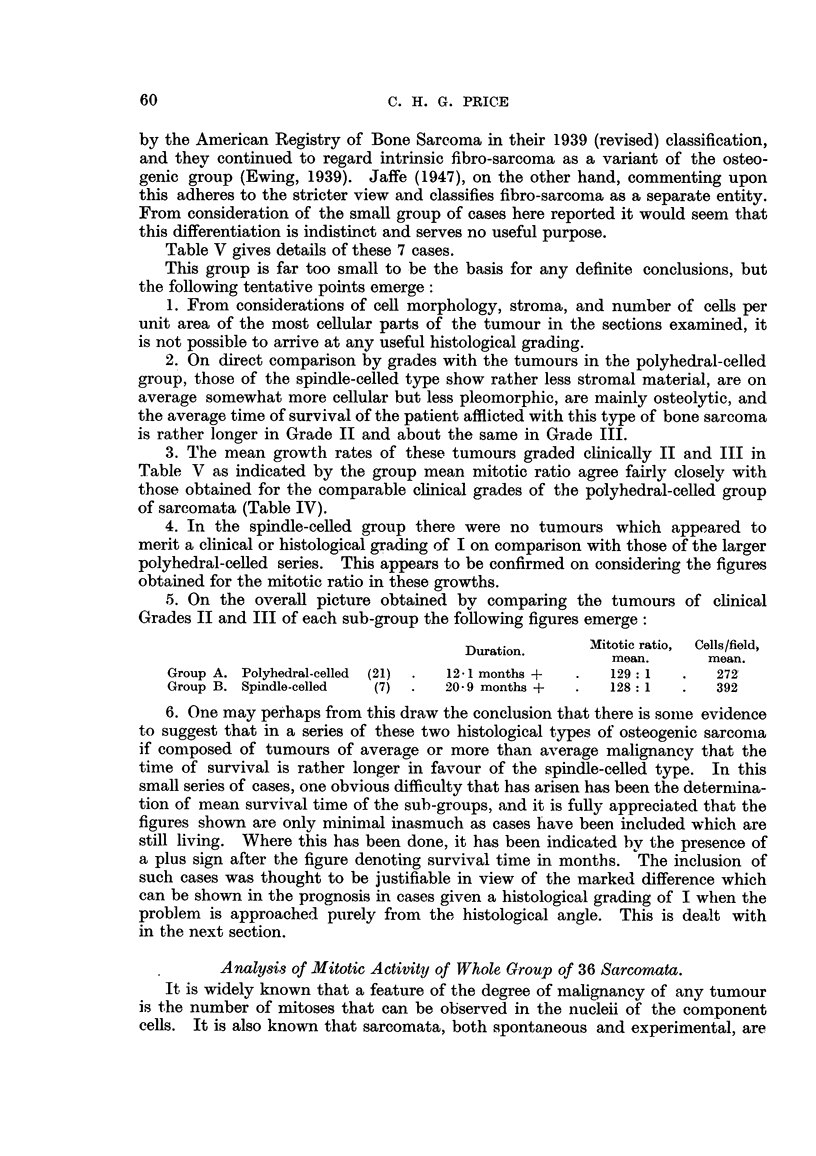

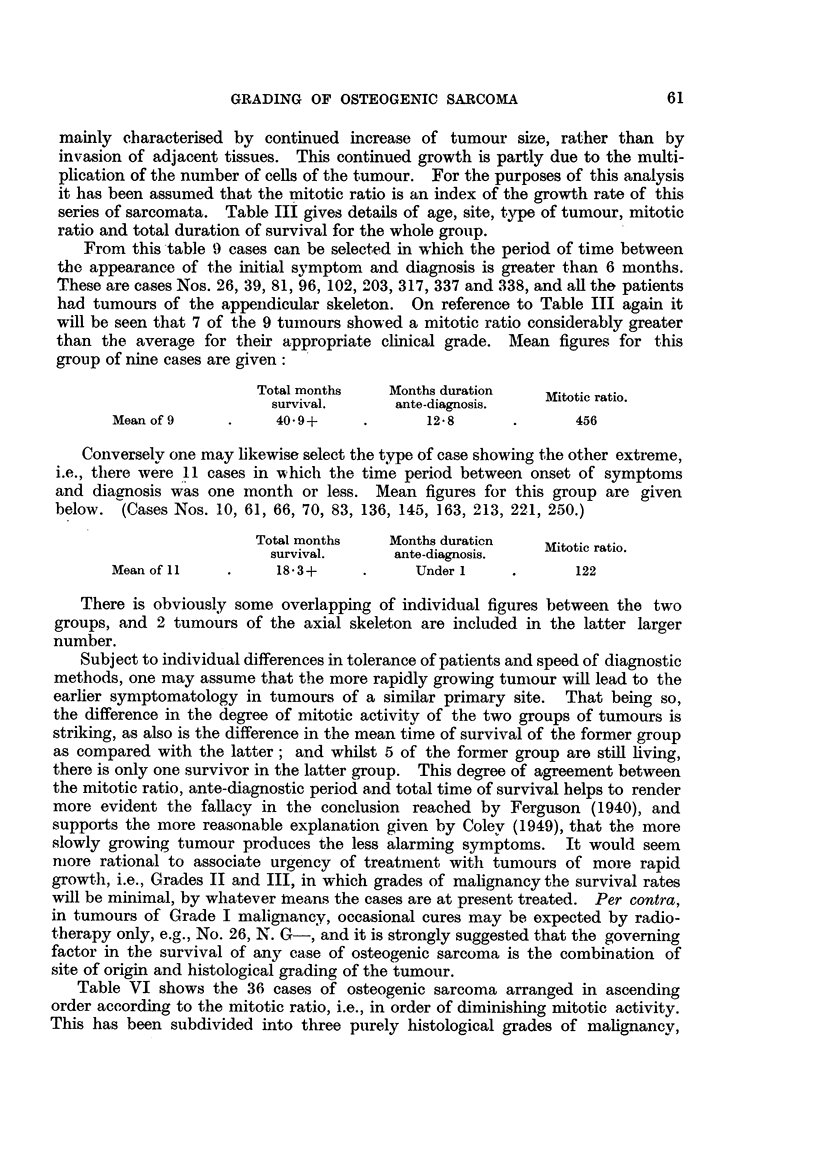

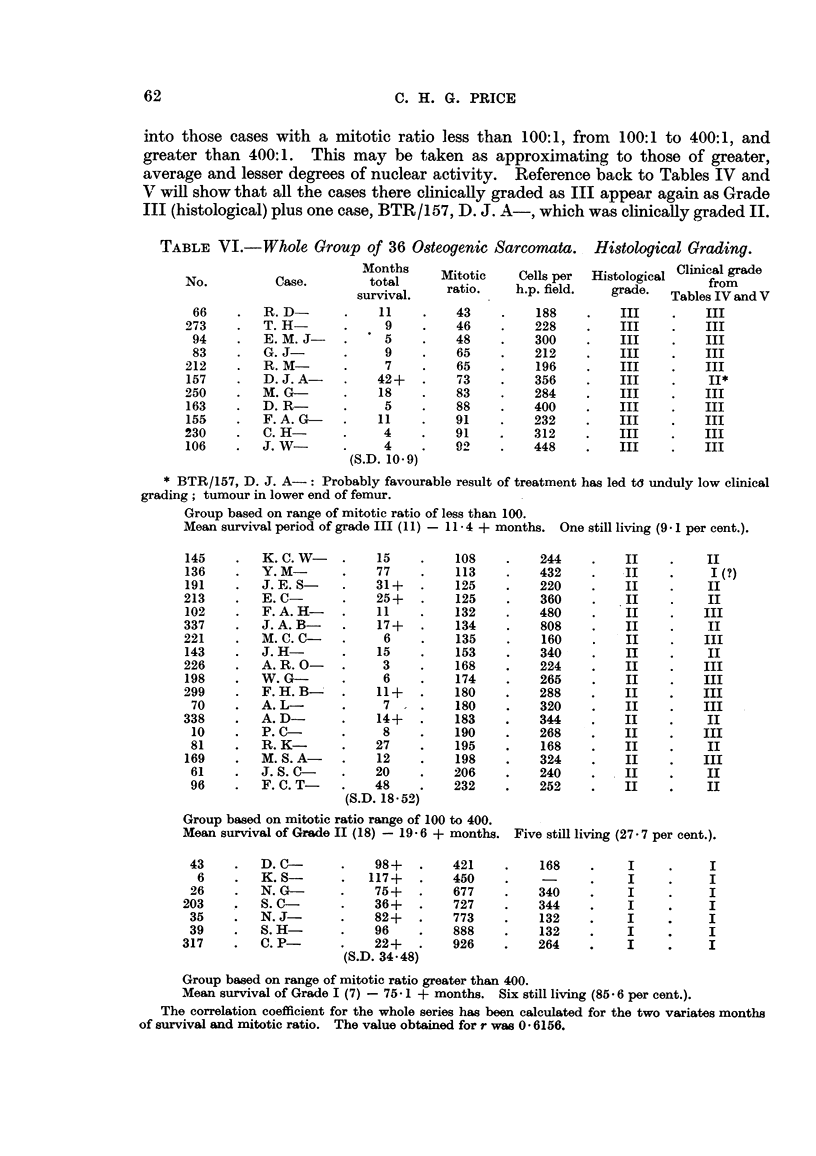

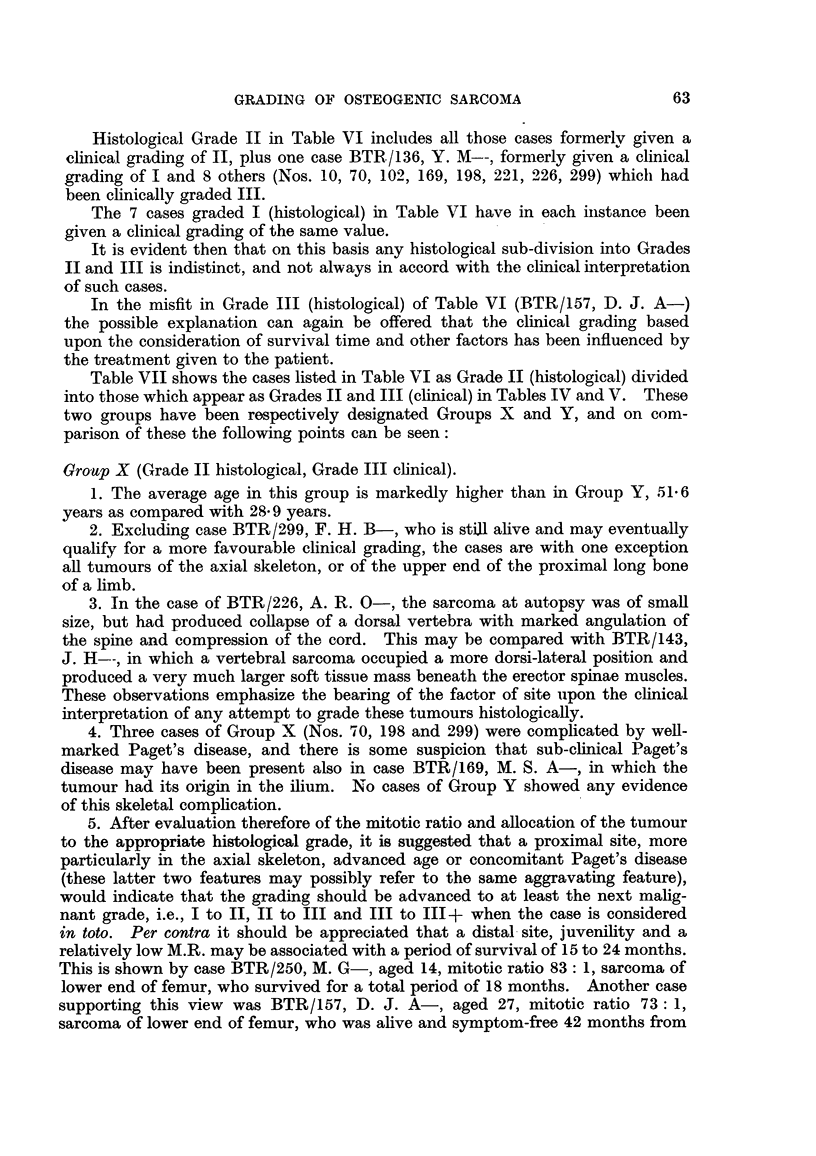

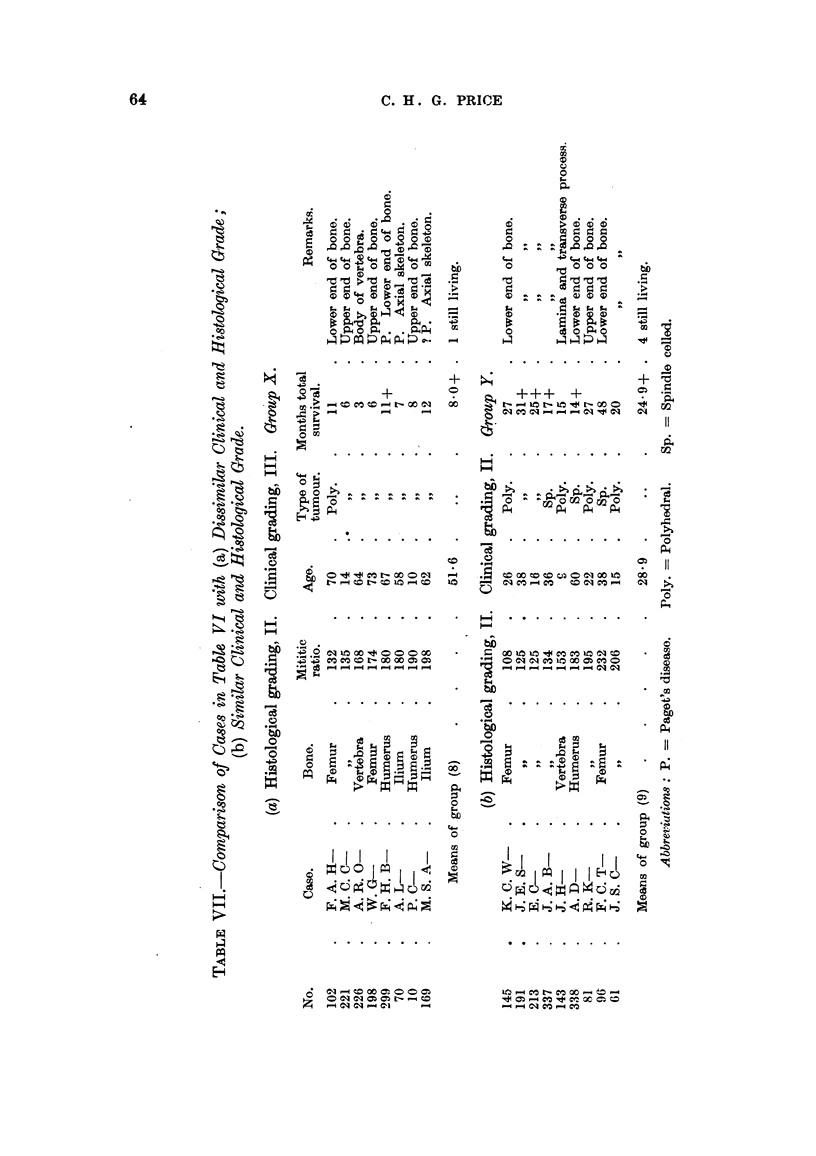

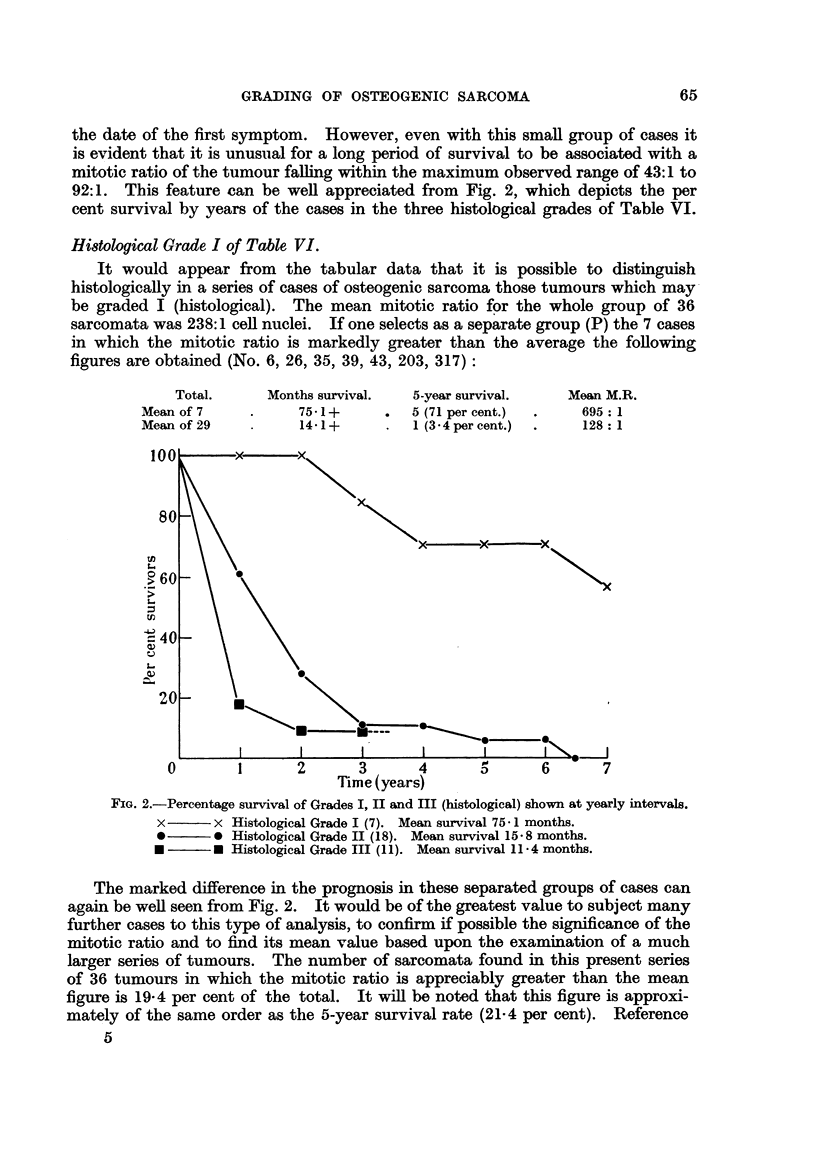

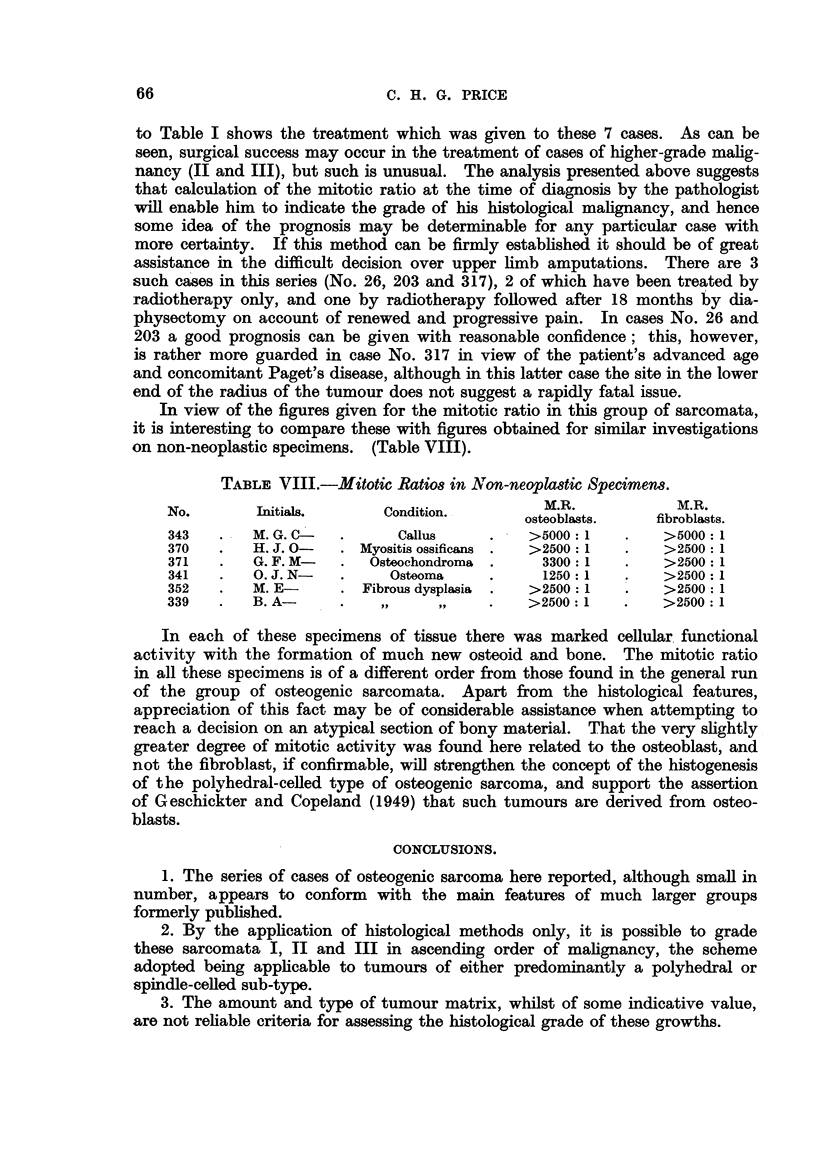

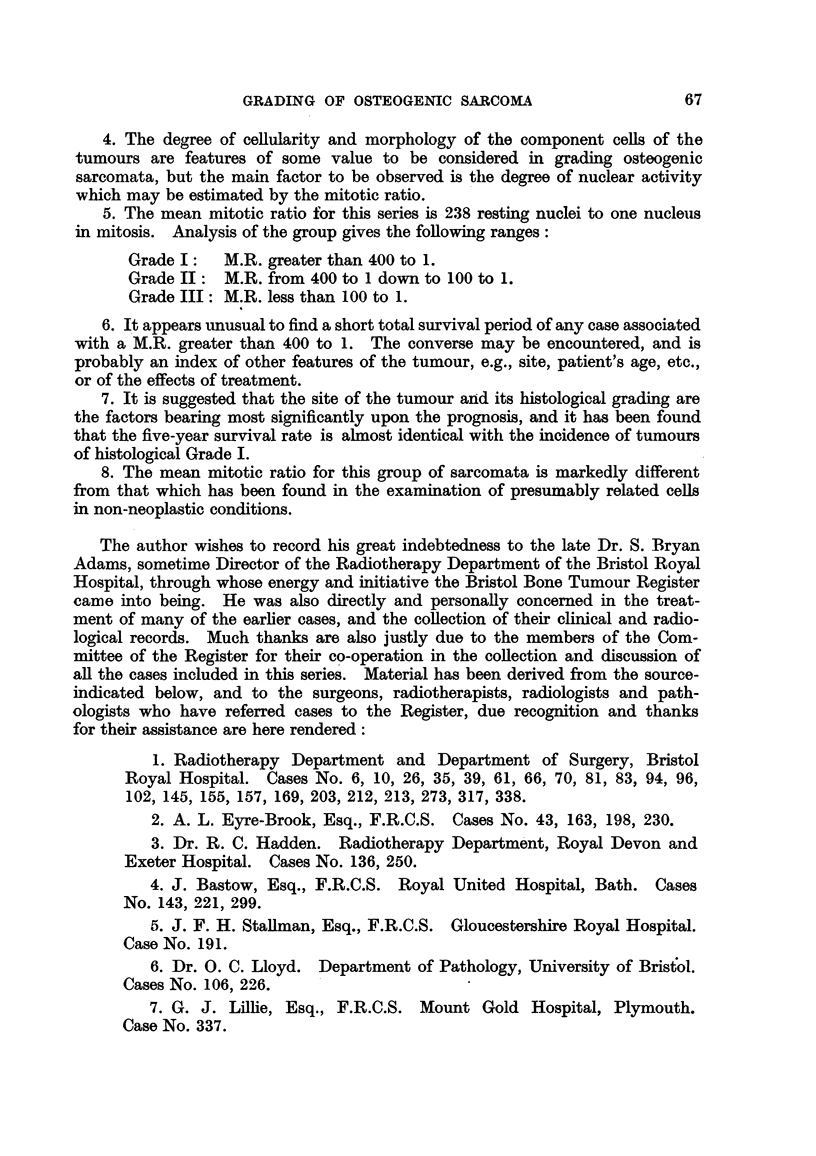

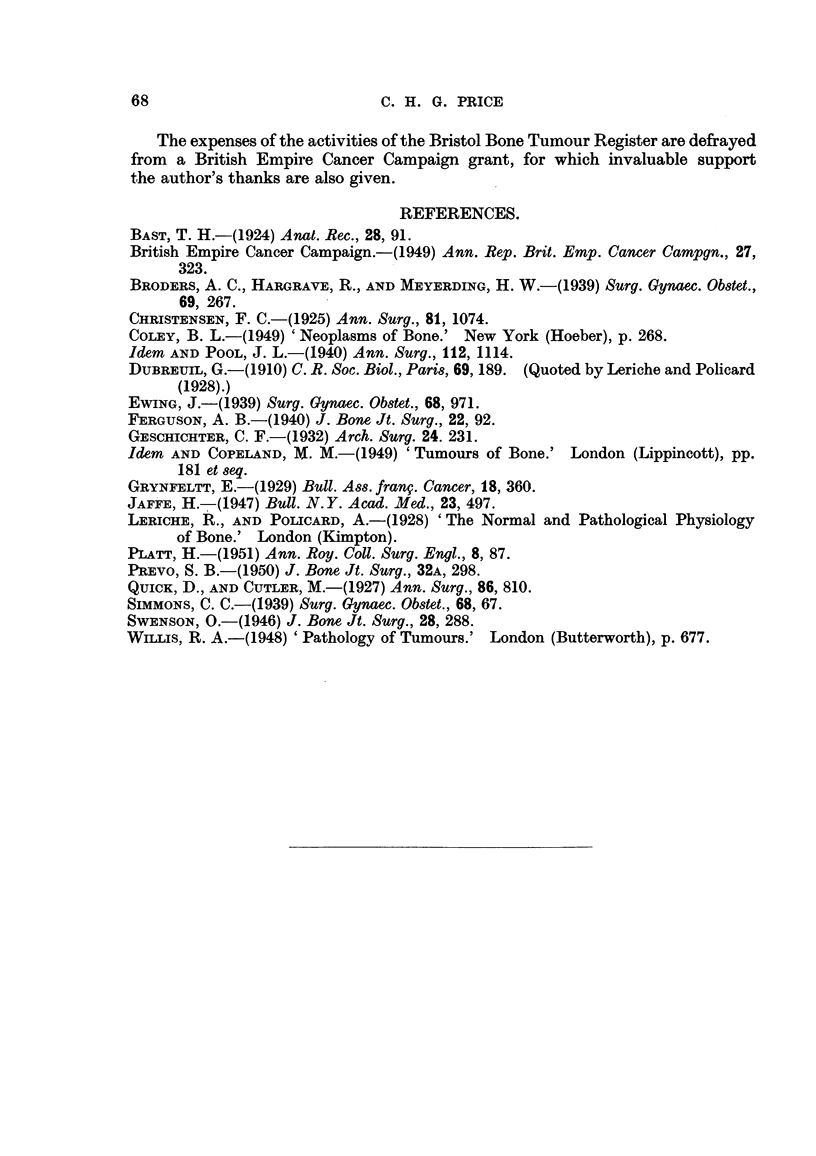

